# Decoding breast cancer treatment resistance through genetic, epigenetic, and immune-regulatory mechanisms: from molecular insights to translational perspectives

**DOI:** 10.20517/cdr.2025.69

**Published:** 2025-07-21

**Authors:** Suryendu Saha, Samikshya Mahapatra, Sinjan Khanra, Barnalee Mishra, Biswajit Swain, Diksha Malhotra, Swarnali Saha, Venketesh K. Panda, Kavita Kumari, Sarmistha Jena, Sandeep Thakur, Pawan K. Singh, Gopal C. Kundu

**Affiliations:** ^1^School of Biotechnology, KIIT Deemed to be University, Bhubaneswar 751024, India.; ^2^School of Applied Sciences, KIIT Deemed to be University, Bhubaneswar 751024, India.; ^3^BVG Life Sciences Ltd., Pune 411034, India.; ^4^Kalinga Institute of Medical Sciences (KIMS), KIIT Deemed to be University, Bhubaneswar 751024, India.

**Keywords:** Breast cancer, drug resistance, genetic mutations, epigenetic factors, biomarkers, immunotherapeutics

## Abstract

Breast cancer continues to be the primary cause of cancer-related deaths among women globally, with increased rates of incidence and mortality, highlighting the critical need for effective treatment strategies. Recent developments have introduced a variety of treatment options that address the molecular diversity of breast cancer; nonetheless, drug resistance remains a significant barrier to achieving favorable results. This review explains the crucial role of genetic and epigenetic changes in contributing to therapeutic resistance, in addition to other factors such as increased drug efflux, enhanced DNA repair, evasion of senescence, tumor heterogeneity, the tumor microenvironment (TME), and epithelial-to-mesenchymal transition (EMT). Genetic modifications, including mutations in oncogenes and tumor suppressor genes, disrupt essential signaling pathways, facilitating resistance to chemotherapy and targeted therapies. At the same time, epigenetic modifications - like DNA methylation, alterations to histones, and dysregulation of non-coding RNAs - reprogram gene expression, supporting adaptive resistance mechanisms. These molecular abnormalities contribute to the plasticity of tumors, allowing cancer cells to evade therapeutic approaches. This review consolidates recent discoveries regarding how these genetic and epigenetic modifications affect treatment responses and resistance in breast cancer, highlighting their interaction with disease advancement. By pinpointing new drug targets, including immunotherapeutic strategies, this article seeks to shed light on the molecular underpinnings of chemoresistance, aiding in the refinement of existing treatment protocols. A more profound understanding of these mechanisms offers the potential for developing precision therapies to overcome resistance, reduce relapse rates, and improve clinical outcomes for breast cancer patients.

## INTRODUCTION

Around 3,10,720 new cases of invasive breast cancer and 56,500 cases of ductal carcinoma *in situ*, as well as a predicted 42,250 fatalities from breast cancer, were expected in the United States alone in 2024, making it a major worldwide health concern^[1]^. Most frequently, breast cancer develops in the milk ducts (ductal carcinoma) or the lobules (lobular carcinoma)^[2]^. There are two types of cancer: *in situ*, which indicates that the cancer has not spread to the rest of the breast, and invasive, which indicates that the cancer has spread to the surrounding breast tissue^[3]^. Invasive ductal carcinomas make up about 72%-80% of all breast cancers^[4]^. Breast cancer can also be categorized into four molecular subtypes according to receptor expression: luminal A, luminal B, HER2-enriched, and triple-negative breast cancer (TNBC). This molecular classification is crucial for administering specific drugs and improving patient outcomes. Endocrine therapy, for instance, is used to treat individuals with tumors that express estrogen receptors; just a minority of these patients also undergo chemotherapy. Tumors that express HER2 receptors are treated with immunotherapy in conjunction with specific targeted therapy. Finally, chemotherapy is the primary form of treatment for individuals with triple-negative malignancies^[5,6]^. Breast cancer is a complex and heterogeneous disease with distinct morphological and molecular features, contrary to a simplistic health concern that involves only a few molecular targets and signaling pathways responsible for progression. According to recent findings, individuals with the same subtype of breast cancer may react differently to a given treatment, indicating the heterogeneous nature of this disease. The major obstacle to lowering the substantial number of deaths from breast cancer in women nonetheless remains overcoming treatment resistance, even after decades of therapeutic advancements^[7]^. To improve patient care and develop effective treatment plans, it is pivotal to comprehend the intricate mechanisms causing drug resistance. Several predominant factors are critical in drug resistance, including DNA repair, loss of cell death, genetic or epigenetic changes, undruggable genomic drivers maintaining oncogenic signaling, therapeutic selections expanding resistant clones, and complex interactions with the immune system and microenvironment.

Breast cancer detection and monitoring are crucial for improving treatment outcomes and patient prognosis^[8]^. Biomarkers are important for effectively diagnosing, predicting, and managing breast cancer^[9,10]^. Many patients require personalized treatments, necessitating the development of novel biomarkers for diagnostics and prognostics, as well as the use of single cells for early cancer diagnosis. Machine learning and artificial intelligence can significantly improve cancer drug development. Cancer biomarkers include biomolecules from cancer cells, which can help assess tumor type, progression, and treatment response^[11]^. Due to tumor cell diversity, a combination of biomarkers is often necessary for accurate diagnosis and monitoring. While some biomarkers are being tested in clinical trials, they still lack adequate sensitivity and selectivity^[12,13]^. Therefore, new, effective biomarkers are essential, particularly for advancing immunotherapies. This review highlights the complexity of drug resistance mechanisms and additionally emphasizes the significance of cutting-edge research in overcoming breast cancer resistance by developing combination therapies, including immunotherapeutics and personalized treatments, and their profound implications for clinical outcomes.

## BREAST TUMOR HETEROGENEITY, STEM CELLS, AND DRUG RESISTANCE

The heterogeneity and complexity of the tumor microenvironment (TME) are shaped by various cellular and subcellular components, contributing to its remarkable plasticity and resulting in therapeutic resistance in breast cancer^[14]^. Tumor heterogeneity is facilitated by genetic mutations, transcriptional and translational variations, as well as epigenetic alterations to these cellular characteristics^[15]^. TME is majorly composed of cancer-associated fibroblasts (CAFs), tumor-associated macrophages (TAMs), cancer stem cells (CSCs), tumor-derived endothelial cells (TECs), cytokines, and various growth factors^[16]^. Among stromal cells, CAFs are a vital component of the TME and are responsible for extracellular matrix (ECM) maintenance and remodeling^[17,18]^. Yuan *et al.* demonstrated that CAFs confer resistance to tamoxifen in breast cancer cells via the GPER-integrin β1-mediated pathway^[19]^. The GPER/EGFR/ERK pathway enhances β1-integrin expression, accelerating CAF-induced epithelial-to-mesenchymal transition (EMT) and leading to resistance to tamoxifen in breast cancer cells^[19]^. Furthermore, CAFs activate numerous signaling pathways, including JAK/STAT3, PI3K/Akt, and NF-κB, to promote trastuzumab resistance in HER2-positive breast cancer cells^[20]^. Gao *et al.* demonstrated that the CD63^+^ CAF subtype contributes to tamoxifen resistance by releasing exosomal miR-22, which leads to the downregulation of ERα and PTEN expression in breast cancer^[21]^. In addition, CD10^+^GPR77^+^ CAFs, TSPAN8^+^ myCAFs, and PDPN^+^ CAFs confer chemoresistance to various drugs in breast cancer^[22-24]^.

TAMs exert dynamic functional attributes by fostering oncogenic response within the TME^[25]^. These cells promote cancer cell proliferation and angiogenesis, while also suppressing immune responses, which aids in tumor progression^[26]^. The breast tumor core comprises specialized cells, such as breast cancer stem cells (BCSCs), which are heterogeneous and distinguished by various surface markers. BCSCs confer resistance in two ways: initially sensitive but gradually developing resistance to anti-cancer therapies and intrinsic drug resistance. Due to their self-renewal and differentiation abilities, these cells can form various cell types within the TME^[27]^. Following chemotherapy, the population of CSCs in the tumor rapidly increases as these cells can survive and expand even after the majority of cancer cells have been eradicated^[28]^. CSCs can withstand therapy primarily because they exhibit multidrug resistance (MDR) transporters, have a robust DNA repair mechanism, and induce stronger apoptotic resistance in contrast to other cells^[29]^. The key surface markers of BCSCs, such as CD44, ALDH1, CD133, EpCAM, ABCG2, GD2, and CXCR4, aid in promoting chemoresistance to major drugs, including paclitaxel, anthracyclines, tamoxifen, fulvestrant, letrozole, exemestane, palbociclib, trastuzumab, and lapatinib^[30]^. HA-CD44 interaction enhances breast cancer MDR by regulating MDR1 through the STAT3 pathway^[31]^. CD133^high^ BCSCs display resistance to hormonal therapy and promote metastasis via IL-6/Notch3 signaling in ER+ breast cancer^[32]^. Another study reveals that doxorubicin sensitivity in BCSCs can be increased by EpCAM aptamer-mediated survivin silencing, reversing resistance in breast cancer^[33]^. ALDH mainly regulates breast tumor progression and metastasis, while inhibition of ALDH activity contributes to the reversal of doxorubicin/paclitaxel resistance in CD44^+^ALDH^high^ BCSCs^[34]^. Hence, exploring the diverse TME components and targeting their associated molecular players may unravel novel therapeutic strategies for breast cancer.

## GENETIC FACTORS INVOLVED IN BREAST CANCER RESISTANCE

Prior studies identified several characteristics of inherited gene mutations that are recognized to be associated with the advancement of breast malignancies. The genes most frequently linked to familial breast cancer, which accounts for 80% of cases, are *TP53*, *BRCA1*, *BRCA2*, *PTEN*, *STK11*, and *CDH1*. Rarely, 2%-3% of these cases are caused by mutations in moderate-penetrance genes such as *BRIP1*, *CHEK2*, *PALB2*, and *ATM*^[35]^. Loss of BRCA1 activity may also be linked to resistance to spindle poisons and susceptibility to chemotherapy that damages DNA^[36]^. The ectopic expression of mutant variants of PTEN, which are either devoid of lipid phosphatase (G129E) or both lipid and protein phosphatase (C124S) activity, results in a reduction in doxorubicin sensitivity in MCF-7 cells with wild-type PTEN while simultaneously increasing sensitivity to the mTOR inhibitor, rapamycin^[37]^. The ABCB1 3435C>T polymorphism has been linked to anthracycline resistance in numerous investigations. For instance, patients with the CT genotype experience poor prognosis, while those with the TT genotype are associated with a less favorable clinical response^[38,39]^. TP53 mutations, c-erbB-2 expression, Bcl-2 negativity, and high histological grade are linked to doxorubicin resistance in breast cancer subtypes. Some patients with TP53 mutations respond to treatment, indicating that other factors may contribute to resistance^[40]^. A previous study illustrated a robust connection between resistance to 5-fluorouracil, mitomycin, and mutations in the p53 protein. These alterations could develop resistance against multiple chemotherapeutic drugs in the management of breast cancer^[41]^. Drug resistance is linked to p53 mutations, namely those that impact the L2/L3 domains. Most significantly, CHEK2 mutations that produce a non-functional protein have been associated with drug resistance *in vitro*^[42]^. The mouse double minute 2 (*MDM2*) gene encodes the MDM2 protein, which is the primary negative regulator of the p53 protein. The overexpression of MDM2 causes tumor cells to undergo EMT, which makes them resistant to chemotherapy^[43]^. In HER2-positive breast cancer, resistance to trastuzumab regimens is linked to the overexpression of MDM2^[43]^. To initiate ATM activity, zinc finger E-box binding homeobox 1 (ZEB1) binds to the ATM promoter, forming a ZEB1/p300/PCAF complex. ZEB1 expression rises in breast cancer and is positively correlated with levels of ATM protein. Under *in vitro* and *in vivo* conditions, ZEB1 downregulation makes breast cancer cells more susceptible to chemotherapy^[44]^.

## EPIGENETIC MECHANISMS IMPARTING DRUG RESISTANCE IN BREAST CANCER

**Figure 1:**
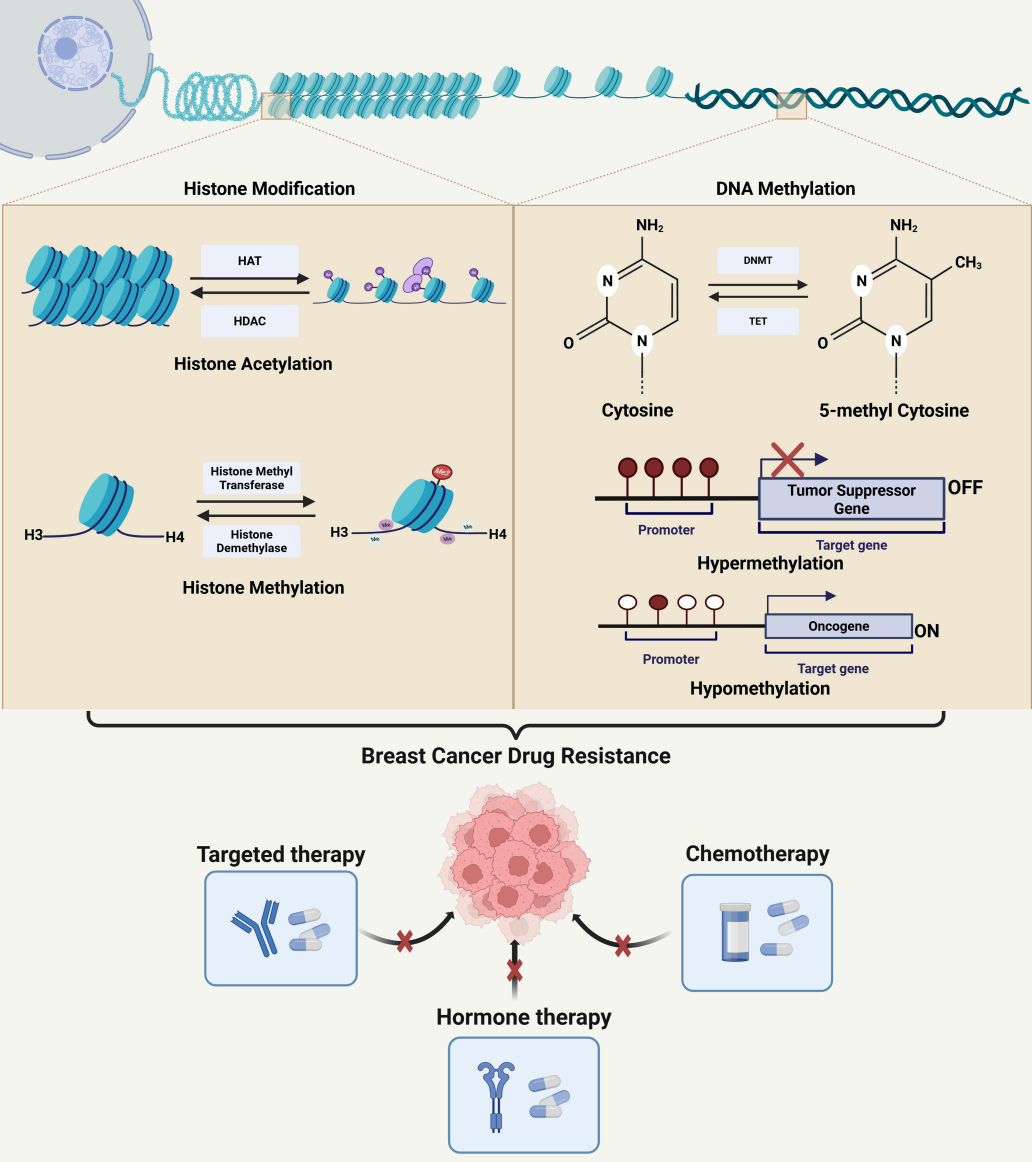
Overview of key epigenetic mechanisms conferring drug resistance in breast cancer. This illustration highlights how epigenetic modifications significantly promote drug resistance in breast cancer. Breast cancer gene expression patterns are regulated by epigenetic changes such as DNA methylation and histone modification that remold chromatin structure and DNA accessibility. DNMT and DNA demethylase (TET: Ten-eleven translocation family proteins) are responsible for maintaining DNA methylation. Acetylation and methylation are the two fundamental ways that histones can be modified. Histone acetylation is controlled by HAT and HDAC, whereas histone methylation is regulated by histone demethylases and histone methyltransferases. The hypomethylation of oncogenes and the hypermethylation of tumor suppressor genes are important events that significantly impact drug responsiveness and the emergence of resistance by dynamically controlling the expression and functionality of genes essential for drug uptake, metabolism, and target interaction. Created in BioRender. Malhotra, D. (2025) oxxrw2s. DNMT: DNA methyltransferase; HAT: histone acetyltransferase; HDAC: histone deacetylase.

Recent findings have highlighted the significance of epigenetic alterations in developing and sustaining drug resistance in breast carcinoma, offering new avenues for therapeutic intervention^[45]^. Small-molecule drugs can effectively target dysregulated epigenetic pathways, unlike genetic mutations, which are difficult to overcome. Moreover, modification of the epigenome makes cancer cells more prone to immunological attacks and enhances their susceptibility to immunotherapy^[46,47]^. Epigenetic alterations, including histone modification and DNA methylation, aid in the knockdown of tumor suppressor genes and the proliferation of oncogenes, leading to cancer progression and drug resistance^[48,49]^. Figure 1 depicts these epigenetic changes, such as DNA methylation and histone acetylation/methylation, regulated by DNMT, TET, HAT, HDAC, and histone methyltransferases/demethylases, which alter chromatin structure and gene expression in breast cancer. Oncogene hypomethylation and tumor suppressor hypermethylation drive drug resistance by regulating genes involved in drug uptake, metabolism, and target interaction.

### DNA methylation and drug resistance

Gene silencing often results from DNA methylation, which involves the methylation of cytosine residues in CpG islands^[50]^. Aberrant DNA methylation patterns often lead to drug resistance in cancer. These patterns include hypomethylation of oncogenes and hypermethylation of tumor suppressor genes [Figure 1]^[51]^. Zhang *et al.* revealed that ZEB1 causes DNA hypermethylation and ERα downregulation in breast cancer cells via interaction with DNMT3B and HDAC1 at the ERα promoter. Since ZEB1 expression in breast cancer is inversely linked to ERα protein levels, downregulating ZEB1 significantly increases the responsiveness of breast cancer cells to estrogen inhibitor therapy using tamoxifen and fulvestrant under *in vitro* and *in vivo* conditions. Therefore, ZEB1 promotes hypermethylation of the ERα promoter, leading to estrogen inhibitor resistance in breast cancer^[52]^. Another possible mechanism by which tumors develop chemoresistance is through hypomethylation of promoters for particular gene types. Chekhun *et al.* demonstrated that high levels of methylation are observed in the promoter region of several chemoresistance-related genes, including glutathione-S-transferase (GSTπ), multidrug-resistant 1 (MDR1), urokinase, and O(6)-methylguanine-DNA methyltransferase (MGMT) in MCF-7 cells, but not in the MCF-7/R variant^[53]^. This hypomethylation is linked to increased P-glycoprotein (P-gp) expression, which is primarily responsible for the resistance of MCF-7 cells to doxorubicin^[53]^. Ansar *et al.* indicated that hypomethylation of the TSTD1 promoter results in an elevated expression of the *TSTD1* gene. Furthermore, the overexpression of TSTD1 in MCF-7 cells contributes to enhanced resistance to hormone therapy using tamoxifen and chemotherapy with epirubicin and docetaxel^[54]^.

### Histone modifications

Histone modifications, including acetylation, methylation, phosphorylation, and ubiquitination, influence gene expression and chromatin architecture. According to Liu *et al.*, acetylated MORC2 binds to histone H3 phosphorylated at threonine 11 (H3T11P), which is necessary for the transcriptional suppression of its downstream target genes, *Cyclin B1* and *CDK1*, as well as for reducing H3T11P levels in response to DNA damage^[55]^. This helps to activate the G2 checkpoint and offers a potential therapeutic approach to make breast cancer cells more sensitive to chemotherapy and radiation therapy^[55]^. He *et al.* revealed that suppressing miR-320a expression leads to the upregulation of TRPC5 and NFATC3, which are essential for chemoresistance in breast cancer^[56]^. This change occurs due to the methylation of the miR-320a promoter and hypomethylation of the ETS-1 promoter, impacting the regulation of TRPC5 and NFATC3^[56]^. LINC00115 is a novel epigenetic modulator of drug-resistant BCSC that leads to the activation of HIF1α signaling through the ALKBH5/YTHDF2 and SETDB1/PLK3 networks. Additionally, methylation at PLK3 K106/K200 enhances the stability of HIF1α and the characteristics of BCSCs, and it also serves as an indicator for individuals with metastatic breast cancer^[57]^.

### RNA modifications

RNA modifications, collectively known as the epitranscriptome, are crucial in regulating gene expression and influencing significant cellular processes^[58]^. An essential epigenetic process involves the methylation of mRNA, particularly through N6-methyladenosine (m6A) modifications. These changes affect mRNA splicing, translation, stability, and degradation, thus regulating the expression of genes associated with drug resistance. For example, m6A modifications have been associated with chemoresistance in breast cancer by modulating mRNA processing, stability, and protein synthesis involved in enhanced drug efflux and reduced apoptosis^[59]^. In tamoxifen-resistant MCF-7 cells, the addition of m6A to adenylate kinase 4 (AK4) mRNA increases its expression, subsequently leading to elevated levels of reactive oxygen species (ROS) and p38. Conversely, reducing METTL3 and AK4 expression resensitizes these cells to tamoxifen^[60]^. Wang *et al.* demonstrated that lncRNA A1BG-AS1 promotes adriamycin resistance in breast cancer cells by recruiting the m6A reader, Insulin-like growth factor 2 mRNA-binding protein 2 (IGF2BP2), to stabilize ABCB1 mRNA, enhancing drug efflux and reducing apoptosis^[61]^. RNA editing at 26 locations within the 3′UTR influences the expression of dihydrofolate reductase, leading to the development of highly proliferative breast cancer cells that exhibit resistance to methotrexate^[62]^.

## EPIGENETIC MODIFICATIONS OF NON-CODING RNAs ASSOCIATED WITH BREAST TUMOR CHEMORESISTANCE

Non-coding RNAs (ncRNAs), which encompass microRNAs (miRNAs), LncRNAs, PIWI-interacting RNAs (piRNAs), and circular RNAs (circRNAs), are crucial in regulating tumor progression and influencing resistance to therapy due to their lack of protein-coding functions. In breast cancer (BC), the epigenetic alterations of ncRNAs - which encompass DNA methylation, RNA methylation (especially m6A), and histone modifications - significantly impact oncogenic signaling pathways via essential regulatory enzymes such as methyltransferases, acetylases, and ubiquitin-related enzymes^[63-65]^. These modifications regulate gene expression, contributing to tumor advancement and resistance to therapy.

### Regulation of DNA methylation by ncRNA

ncRNAs play an active role in the modulation of DNA methylation by attracting DNMTs to the promoters of target genes. For example, the oncogenic lncRNA TINCR brings DNMT1 to the promoter of miR-503-5p, resulting in hypermethylation and decreased expression, which in turn leads to increased STAT3 levels and promotes breast cancer progression^[66]^. Interestingly, STAT3 accumulates at the promoter of TINCR, creating a positive feedback loop. Similarly, TINCR targets miR-199a-5p, lowering its expression through DNMT1-mediated methylation^[67]^. LncRNA H19 reduces S-adenosine homocysteine hydrolase (SAHH), which in turn diminishes DNMT3B-mediated methylation of the Beclin1 promoter, thereby promoting autophagy and resistance to tamoxifen^[68]^. Furthermore, ncRNAs are themselves influenced by DNA methylation. Genome-wide studies have revealed that methylation patterns undergo significant alterations in breast cancer cells that are resistant to chemotherapy, which aligns with differences in the expression of miRNAs and their target genes. For example, in chemoresistant cells, the promoter of miR-320a is hypermethylated, which affects its ability to target TRPC5 and NFATC3 - crucial factors involved in drug resistance^[56,69]^. This underscores how the epigenetic silencing of miRNA promoters through DNA methylation can sustain oncogenic signaling. Concurrently, lncRNAs such as MEG3 and H19 have been shown to be epigenetically silenced in chemoresistant cell types. The tumor-suppressive lncRNA, MEG3 is silenced through hypermethylation mediated by DNMT1, but treatment with 5-AzadC reverses this methylation and restores its expression, inhibiting Notch1 signaling^[70]^. UXT, in association with DNMT3B, methylates MEG3, diminishing its expression; however, knockdown of UXT leads to an upregulation of both MEG3 and p53^[71]^. In contrast, lncRNA, H19 works in conjunction with EZH2 (a histone-lysine N-methyltransferase enzyme) and attracts DNMTs to methylate the promoters of pro-apoptotic genes, such as *BIK*, thereby silencing these genes and contributing to drug resistance^[72]^. Another lncRNA, MIR497HG, is suppressed by ZEB1-mediated recruitment of DNMT3B and HDAC1/2 in endocrine-resistant breast cancer; meanwhile, ERα enhances its expression in sensitive conditions, affecting the PI3K/Akt pathway via miR-195/497^[73]^.

### RNA methylation (m6A) and ncRNA crosstalk

The interaction between RNA methylation - particularly m6A - and ncRNAs is increasingly acknowledged as a crucial epigenetic factor in the chemoresistance of breast cancer. This relationship governs RNA stability, translation, and splicing, significantly affecting how cells adapt to chemotherapy. m6A regulators such as METTL3 and YTHDF2 influence the stability and activity of lncRNAs and miRNAs, thereby affecting drug sensitivity. For example, the lncRNA, AGAP2-AS1, which is overexpressed in trastuzumab-resistant breast cancer, is stabilized via m6A methylation mediated by METTL3-YTHDF2, boosting its oncogenic role and leading to treatment failure^[74]^. Some lncRNAs, such as lncRNA-CDC6, act as scaffolds that connect METTL enzymes to target RNAs, thereby regulating m6A addition across numerous transcripts that play roles in drug efflux and apoptosis^[75]^. In TNBC, m6A-modified ncRNAs, including specific lncRNAs and miRNAs, contribute to resistance against anthracyclines and taxanes. These modified ncRNAs influence EMT, stemness, and DNA repair - processes closely associated with resistance phenotypes^[76]^. A broader perspective indicates that ncRNAs methylated by m6A frequently show context-dependent effects within the TME, enhancing resistance through modified immune responses, regulation of autophagy, and adaptation to oxidative stress^[77,78]^.

Moreover, the m6A “reader” RNA-binding protein HNRNPA2/B1 has been found to be significantly elevated in tamoxifen-resistant breast cancer LCC9 cells. Comprehensive genome-wide miRNA profiling indicates that the overexpression of HNRNPA2/B1 leads to considerable alterations in various miRNAs, influencing several signaling pathways linked to endocrine therapy resistance, including TGFβ signaling^[79]^. Collectively, these observations underscore the essential roles of m6A-ncRNA interactions in the mechanisms contributing to therapy resistance in breast cancer.

### Histone modifications and ncRNA regulation

Histone modifications orchestrate gene expression changes that promote chemoresistance. In breast cancer, EZH2-mediated suppression of growth-regulating estrogen receptor binding 1 (GREB1), an ERα cofactor, drives tamoxifen resistance^[80]^. Loss of ten-eleven translocation 2 (TET2) reduces ERα expression, fostering endocrine resistance^[81]^. Histone acetyltransferase lysine acetyltransferase 2A (KAT2A) enhances tamoxifen resistance by destabilizing p53 and upregulating amplified in breast cancer 1 (AIB1)^[82]^. Histone demethylase, LSD1 regulates breast CSC self-renewal, contributing to chemoresistance, and interacts with protein kinase C θ (PKC-θ) to modulate EMT, further promoting drug resistance^[83]^. KDM4A governs CSC growth and self-renewal, facilitating therapeutic resistance. Additionally, KDM5A activates insulin-like growth factor 1 receptor (IGF1R) and erythroblastic oncogene B (ERBB) signaling, triggering the PI3K/Akt/mTOR pathway and conferring tamoxifen resistance^[84]^. Conversely, overexpression of peptidyl arginine deiminase 4 (PAD4) induces apoptosis and elevates GSK3β and p53 levels, enhancing adriamycin sensitivity^[85]^.

These epigenetic alterations in ncRNAs contribute to the progression of breast cancer chemoresistance by influencing crucial signaling pathways, presenting possible targets for therapy.

## MOLECULAR CROSSTALK OF MULTIFACETED DRUG RESISTANCE MECHANISMS

Beyond the impact of ncRNAs, the emergence of treatment resistance in breast cancer shows a more comprehensive integration of numerous signaling pathways. Examining molecular crosstalk improves understanding of how various resistance mechanisms interact and contribute to therapy failure. Overexpression of the PI3K/Akt signaling mechanism in breast cancer results in chemotherapeutic resistance, as it interacts with other pathways such as MAPK and Wnt/β-catenin^[86,87]^. MDR proteins, including P-gp and breast cancer resistance protein (BCRP), along with phosphorylated Akt, have been observed in adriamycin- and paclitaxel-resistant MCF-7 cell lines^[88,89]^. Furthermore, enhanced p44/42 expression in the Ras/MAPK cascade has been associated with chemoresistance in breast cancer cells^[90]^. Tamoxifen-resistant tumors show enhanced p38 phosphorylation, which correlates with MAPK signaling activity^[91]^. Trastuzumab-resistant breast cancer cells have been observed to exhibit a consistent upregulation of the TGF-β signaling cascade^[92]^. Palomeras *et al.* demonstrated that genes such as transforming growth factor beta-induced (*TGFBI*), C-X-C motif chemokine ligand 2 (*CXCL2*), and solute carrier family 38 member 1 (*SLC38A1*) are consistently hypermethylated and downregulated in trastuzumab-resistant HER2+ breast cancer, with TGFBI showing the highest silencing at both the mRNA and protein levels^[93]^. TGFBI hypermethylation in primary tumors is substantially associated with trastuzumab resistance in patients with HER2+ breast cancer^[93]^.

Activation of the Hippo pathway via YAP/TAZ contributes to the emergence of CSC characteristics in breast cancer cells, which serve as the foundation for tumor initiation, metastasis, resistance to treatment, and recurrence^[94]^. One upstream regulator in the Hippo pathway is Ras-association domain family 1 isoform A (RASSF1A), which interacts with MST1/2 to regulate mitosis and cell death^[95]^. The loss of RASSF1A is associated with the development of paclitaxel resistance in patients with breast and ovarian cancer^[96]^. RASSF1A is epigenetically silenced by the promoter DNA methylation in over 50% of all solid tumors^[97]^. Er *et al.* have demonstrated the significance of Hh signaling in controlling stemness in HER2-positive breast cancer cell lines that are resistant to trastuzumab^[98]^. Hh signaling components Shh and Ptch1 are often methylated in breast cancer-initiating cells. Hh effector proteins such as KIF7 and SUFU are deregulated through miRNA and histone modifications, whereas GLI1 expression increases due to the loss of KMT-SETD7 methylase in breast cancer cells, driving abnormal Hh signaling^[99]^.

It has been shown that adipocyte-secreted IL-6 can activate STAT3 in MCF-7 cells, leading to EMT and CSC enrichment^[100]^. Additionally, the IL-6/STAT3 axis can enhance resistance to tamoxifen and cyclin-dependent kinase 4/6 (CDK4/6) inhibitors^[101,102]^. Xiang *et al.* discovered that melatonin induces Sirtuin 1 expression and Sirtuin 1 deacetylates STAT3, particularly at K685 in breast cancer xenografts^[103]^. This suggests that melatonin inhibits STAT3-mediated paclitaxel resistance in breast cancer. Additionally, dim light at night-mediated disruption of circadian melatonin via DNA-methylation can promote drug resistance in breast carcinoma^[103]^. Epigenetic alterations in particular genes have also been linked to endocrine resistance. Resistance to endocrine therapy has been specifically linked to decreased expression of Spalt-like transcription factor 2 (SALL2), which in turn causes ERα and phosphatase and tensin homolog (PTEN), which are known modulators of the Akt/mTOR signaling, to be downregulated^[104]^. RTK-RAS signaling hyperactivation and aberrant activation of a CDK4/6 downstream effector, CCNE1-CDK2, restore RB phosphorylation and promote resistance, while also reducing responsiveness to palbociclib^[105]^. EGFR/HER2 activation could be the driving mechanism behind the CSC enrichment in tamoxifen-resistant breast cancer^[106,107]^. Nami *et al.* demonstrated that *ERBB2* gene silencing via epigenetic modulation during EMT could be a mechanism of resistance to lapatinib and trastuzumab in HER2-positive breast cancer cells^[108]^.

**Figure 2:**
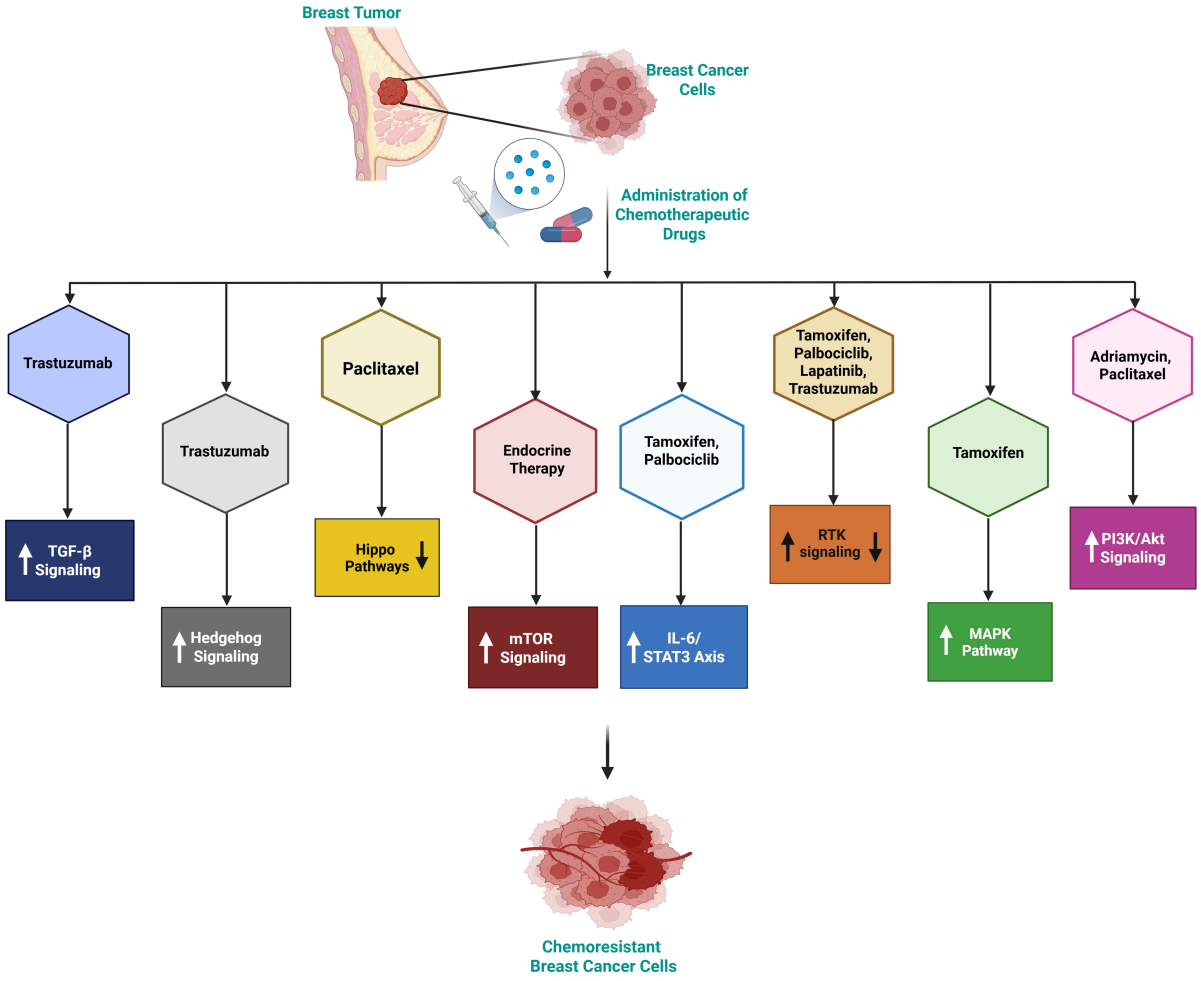
Schematic representation of chemotherapeutic drugs and the signaling pathways involved in resistance mechanisms in breast cancer. The figure demonstrates the major signaling pathways implicated in breast cancer chemoresistance and tumor growth, including the PI3K/Akt, mTOR, MAPK, RTK, IL-6/STAT3, TGF-β, Hedgehog, and Hippo signaling cascades. Epigenetic modifications alter these signaling networks, resulting in EMT, CSC enhancement, and reduced treatment sensitivity. Paclitaxel resistance involves activation of the PI3K/Akt pathway and suppression of Hippo signaling. Endocrine resistance is associated with enhanced mTOR activity. Tamoxifen resistance features elevated IL-6/STAT3 and MAPK signaling. In palbociclib resistance, both IL-6/STAT3 and RTK pathways are upregulated. RTK signaling is also reduced in lapatinib resistance, while PI3K/Akt is enhanced in adriamycin resistance. Created in BioRender. Malhotra, D. (2025) rf3yj6x. EMT: Epithelial-to-mesenchymal transition; CSC: cancer stem cell.

Figure 2 illustrates the key signaling pathways involved in therapeutic resistance, including TGF-β, Hedgehog, Hippo signaling, mTOR, IL-6/STAT3, RTK, MAPK, and PI3K/Akt. These pathways are regulated by epigenetic modifications such as DNA methylation, ncRNA-mediated silencing, and histone modifications, resulting in CSC enrichment, EMT, and therapeutic failure. The crosstalk between these signaling networks promotes tumor survival, growth, and resistance to chemotherapy, endocrine, and targeted therapy. The figure underlines the intricacy of resistance mechanisms and the significance of combination therapies in improving treatment outcomes.

## DNA REPAIR AND DAMAGE RESPONSE GUIDING THERAPEUTIC RESISTANCE

Various DNA repair mechanisms are employed by cancer cells to combat drug-induced DNA damage, enhancing their resistance to therapy and promoting their survival. Due to the vast range of DNA lesion types, several distinct DNA repair pathways have been studied. The base excision repair (BER), mismatch repair (MMR), and nucleotide excision repair (NER) pathways are triggered to fix single-strand breaks (SSBs), whereas the non-homologous end joining (NHEJ) and homologous recombination (HR) pathways fix double-strand breaks (DSBs)^[109]^. Because cancer cells possess a significant ability to withstand anti-cancer treatments and adapt, the DNA damage response (DDR) becomes impaired, which can result in either heightened sensitivity to genotoxic agents or the development of resistance in cancer cells. A faulty DDR facilitates the emergence of tumor heterogeneity by preselecting subclones that exhibit either inherent or acquired resistance, thereby promoting cancer progression and tumor recurrence^[110]^. LRH1 levels are elevated in primary breast cancer tissues from patients who experience early recurrence, as well as in adriamycin-resistant breast cancer cell lines. Increasing LRH1 levels diminishes the efficacy of chemotherapeutic agents such as adriamycin and cisplatin by inhibiting DNA damage, whereas reducing its expression heightens DNA damage. LRH1 facilitates DNA repair and boosts the expression of MDC1, which is associated with chemoresistance. This indicates that targeting the LRH1-MDC1 signaling pathway could aid in the treatment of chemotherapy-resistant breast cancer^[111]^. In breast cancer cells resistant to palbociclib, re-sensitization to the drug is achieved by combining inhibitors against PARP and STAT3. This indicates that concomitant targeting of DDR mechanisms and the IL-6/STAT3 pathway may effectively overcome acquired resistance to palbociclib^[102]^. VX-970, a small-molecule inhibitor of ataxia telangiectasia and Rad3-related (ATR) kinase, has demonstrated radiosensitizing effects in PDX models and TNBC cells. In particular, this molecule decreases colony formation after radiation treatment in TNBC cells, prolongs DNA DSBs, and inhibits the signaling of the ATR-CHK1-CDC25a axis. Compared to normal epithelial breast cells, these effects are specific to cancer cells^[112]^. One of the primary enzymes in the BER pathway is the AP endonuclease 1 (APE1). In human breast cancer cell lines, lucanthone, an inhibitor of topoisomerase II and APE1 endonuclease, has been demonstrated to enhance the cytotoxic action of alkylating drugs^[113]^.

**Figure 3:**
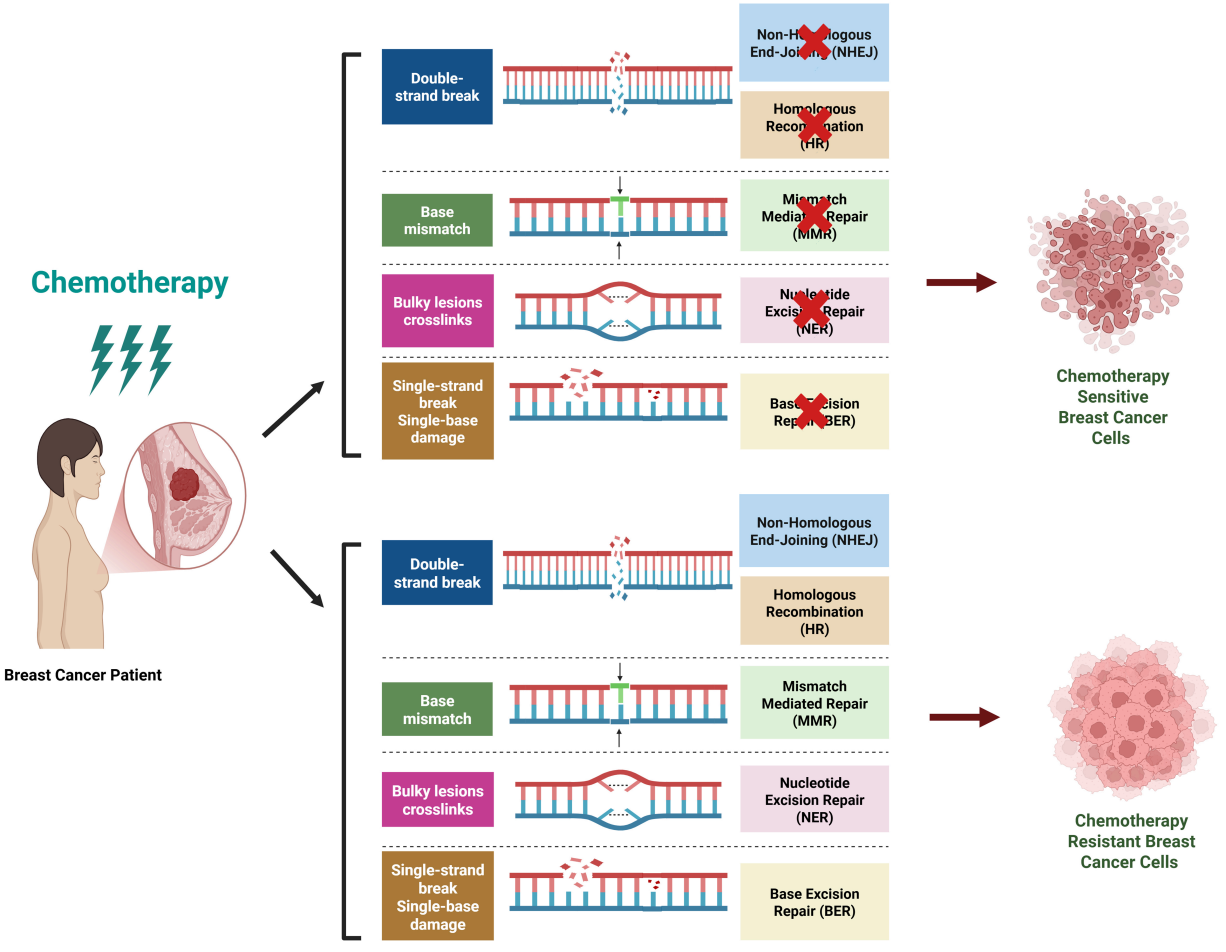
Chemotherapy-induced DNA damage repair in breast cancer, highlighting sensitivity and resistance mechanisms. In chemotherapy-sensitive cells, treatment induces DNA damage mechanisms such as DNA crosslinking, DSBs, and base modifications, leading to impaired DNA repair and subsequent cell death. In contrast, chemotherapy-resistant cells activate robust DNA repair mechanisms, including NHEJ, BER, NER, MMR, and HR, enabling them to effectively repair DNA damage and thereby promote survival. Created in BioRender. Malhotra, D. (2025) m6nst1k. DSBs: Double-strand breaks; NHEJ: non-homologous end joining; BER: base excision repair; NER: nucleotide excision repair; MMR: mismatch repair; HR: homologous recombination.

Figure 3 elaborates how chemotherapy-sensitive cells undergo DNA damage (crosslinking, DSBs, base modifications) with impaired repair, leading to cell death, whereas resistant cells activate robust DNA repair mechanisms (NHEJ, BER, NER, MMR, HR), effectively repairing damage and promoting survival.

## INHIBITION OF APOPTOSIS AND ACQUISITION OF DRUG RESISTANCE IN BREAST CANCER

Malignant cells evade apoptosis through abnormal protein expression, leading to uncontrolled growth and resistance in cancer cells^[114]^. Inhibitors of apoptosis proteins (IAPs), p53 mutations, the Bcl-2 family, and the proteins involved in the PI3K/Akt pathway are among the proteins that are fundamentally engaged in preventing the onset of apoptosis to develop resistance mechanisms^[114]^.

Overexpression of IAPs, including X-linked inhibitors of apoptosis protein (XIAP), inhibits caspase activation in breast cancers, thereby facilitating the escape from apoptosis and the acquisition of resistance^[115]^. Mutant p53 fails to trigger apoptosis, causing resistance to chemotherapy^[116]^. Another important facet of cell survival is the alteration of the PI3K/Akt pathway, which protects cells from apoptosis by phosphorylating many apoptotic proteins^[117]^. By activating NF-κB and phosphorylating IKK, the PI3K/Akt pathway inhibits caspases and ceases apoptosis by upregulating XIAP and Survivin, leading to resistance and relapse^[118]^. The upregulation of cellular FLICE-inhibitory protein (c-FLIP) suppresses the extrinsic mode of apoptosis in CSCs by interfering with Caspase-8 (CASP8) activation, hindering apoptosis^[119]^. TRAIL, FasL, and Granzyme B-induced apoptosis is reduced when miR-519a-3p is overexpressed. miR-519a-3p directly blocks key apoptotic proteins such as CASP8, Caspase-7 (CASP7), TRAILR2 (TNFRSF10B)^[120]^. Autophagy-related proteins, such as Beclin-1, prevent caspase activation and the production of apoptotic complexes, thereby allowing cancer cells to evade cell death^[121]^. Dysregulation of these apoptotic pathways promotes tumor formation, survival, and resistance to therapy.

Advances in therapeutic strategies have improved our knowledge of the molecular processes associated with apoptosis resistance. Emerging therapies exhibiting the potential to overcome resistance include autophagy inhibitors, epigenetic modulators, BH3 mimetics, and SMAC mimetics. ABT-737 and Venetoclax are examples of BH3 mimetics that target the Bcl-2 family to combat apoptosis inhibition^[122,123]^. Additionally, CHK1 inhibitors specifically target chemotherapy-resistant tumor cells, epigenetic modulators such as HDAC inhibitors enhance chemotherapy response, and restoring miRNA-34a increases chemotherapy sensitivity in resistant cells as well^[124-126]^.

## BIOMARKERS IN PREDICTING THERAPY FAILURE

Biomarkers are categorized into diagnostic, prognostic, and predictive subtypes, associated with biological disorders to achieve favorable clinical outcomes and therapeutic success. A diagnostic biomarker detects or confirms a disease, a predictive biomarker identifies individuals likely to respond favorably or unfavorably to treatment, and a prognostic biomarker indicates the likelihood of disease recurrence or progression^[11]^. Yang *et al.* have shown that S100 calcium-binding protein P (S100P) and hyaluronoglucosaminidase 2 (HYAL2) may serve as biomarkers for diagnosing early-stage breast cancer, using the HumanMethylation27 BeadChip^[127]^. In a previous study, elevated levels of plasma-derived exosomal miR-21 and miR-1246 have been reported as indicators of breast cancer^[128]^. Another report showed that IL-6 induces STAT3-mediated miR-21 activation, and functions as an epigenetic switch in breast cancer^[129]^. miRNAs such as miR-145 and miR-21, when deregulated, can be used to differentiate between cancerous and non-cancerous conditions. miR-145 is downregulated, whereas miR-21 is upregulated in breast cancer^[130]^. At present, PD-L1 is one of the approved predictive biomarkers for immune therapeutics in TNBC^[131]^. According to Lei *et al.*, PD-L1 is regulated by the FGFR2-BRD4 axis, which controls the transcriptional network of histone 3 modification in TNBC^[132]^. Another study revealed that a higher expression of WEE1 is associated with resistance to Olaparib, a PARP inhibitor, in breast cancer patient-derived xenograft models^[133]^. It was reported that TNBC patients receiving neoadjuvant paclitaxel exhibit overexpression of miR-18a, which is associated with inhibition of Dicer expression and increased resistance to paclitaxel^[134]^. In ER+ breast cancer cell lines, elevated levels of miR-18a modulate cell polarity, leading to activation of the Wnt pathway and actin remodeling, which may underlie the poor prognosis observed in ER+ breast tumors in clinical settings^[135]^. CircCDYL2 is identified to be upregulated in a significant way in trastuzumab-resistant HER2-positive breast cancer cells and is currently being investigated as a potential diagnostic biomarker^[136]^. In a previous study, it was shown that CDYL2 recruits G9a and EZH2 to epigenetically repress miR124, which promotes NF-κB and STAT3 activation and negatively impacts the prognosis of breast cancer^[137]^. The upregulation of ATP citrate lyase (ACLY) increases the expression of the resistant proteins such as ABCB1/ABCG2, making breast cancer cells resistant to docetaxel, thereby suggesting ACLY as a potential predictive biomarker of tumor recurrence in breast cancer^[138]^. Unc-51-like autophagy activating kinase 1 (ULK1), recognized as a target of NSD2-mediated histone H3K36me2 methylation, promotes activation of ULK1 transcription in TNBC cells. Moreover, ULK1 upregulation predicts an unfavorable clinical outcome for the TNBC patients^[139]^. Therefore, identifying robust biomarkers, such as molecular signatures and ncRNA profiles, is pivotal for predicting therapy failure or drug response in breast cancer, guiding personalized treatment strategies; future research should focus on integrating multi-omics data and validating these biomarkers in prospective clinical trials to enhance therapeutic efficacy and overcome resistance. The clinical utility of several pertinent breast cancer biomarkers is elaborated in Table 1.

**Table 1:** The clinical significance of major breast cancer biomarkers

**Type of biomarkers**	**Name of biomarker**	**Source**	**Validation status**	**Clinical relevance**	**Therapeutic application**	**Ref.**
Molecular biomarkers	SF3B3	Tumor tissue, fulvestrant-resistant and tamoxifen cross-resistant LCC9 and tamoxifen-resistant LCC2 cells	Primarily preclinical research level, early clinical stage	Higher expression of SF3B3 correlates with poor prognosis in ER+ patients, resistance to endocrine therapy (tamoxifen/fulvestrant)	SF3B3 inhibitors effective in preclinical breast cancer models	[140]
	ACLY	Tissue	Primarily preclinical research level, early clinical stage	Recurrence prediction, prognosis and resistance to docetaxel	ACLY inhibitors effective in preclinical breast cancer models; combination with endocrine therapies, chemotherapy	[138,141]
	p27kip1	Tumor tissue	Preclinical validation. Partial clinical evidence	Prognosis, resistance to taxane, endocrine therapy	Enhanced OS of tamoxifen and taxane-treated patients	[142,143]
	TOP2A	Tumor tissue	Clinical, Phase II trial	Prognostic, resistance to adjuvant anthracycline-based chemotherapy	TOP2A-targeted therapies to enhance anthracycline efficacy	[144-146]
	HER2	Tissue	Clinical, Phase II trial	Diagnosis, prognosis, resistance to chemotherapy	Anti-HER2 therapies (e.g., pertuzumab) to overcome resistance	[146,147]
	cGAS-STING	Tumor tissues	Preclinical	Prognosis, resistance to Herceptin	STING agonists to enhance immunotherapy response	[148,149]
	SH3BGRL	Tumor tissue	Preclinical	Diagnostic, resistance to HER2-targeted therapy	Potential target for novel HER2 therapy adjuvants	[150]
	CTMP	Tissue, SK-BR-3 and BT-483 cell line	Preclinical	Prognosis, resistance to trastuzumab	CTMP inhibitors to enhance trastuzumab efficacy	[151]
	Ki67	Tumor tissue	Clinical, Phase II trial	Prognosis, resistance to adjuvant tamoxifen	CDK4/6 inhibitors to overcome tamoxifen resistance	[152,153]
	AR-V7	Tumor tissue, MDA-MB-453, MDA-MB-231, MCF-7	Preclinical	Prognosis, resistance to endocrine therapy	AR inhibitors (e.g., enzalutamide) to target AR-V7	[154]
	FASN	Mammary tumor tissue	Clinical, Phase II trial	Prognosis, resistance to cisplatin	FASN inhibitors to overcome cisplatin resistance in TNBC	[155-157]
	BRCA1 and BRCA2	Tumor tissue	Clinical, Phase III trial	Prognosis, resistance to taxane	PARP inhibitors (e.g., olaparib) for BRCA-mutated tumors	[158]
	SMAD4	Tumor tissue	Preclinical	Prognosis, resistance to endocrine therapy	SMAD4 regulating 4-hydroxytamoxifen sensitivity in breast cancer	[159]
	Cyclin D1	Tumor tissue	Preclinical	Prognosis, resistance to hormonal therapy	CDK4/6 inhibitors (e.g., palbociclib) to target Cyclin D1	[160]
	TP53	Tumor tissue	Clinical, Phase III trial	Prognosis, resistance to preoperative endocrine therapy (tamoxifen, aromatase inhibitors)	TP53-targeted therapies or combination strategies	[161,162]
	VEGF-A	Tumor tissue	Clinical, Phase III trial	Diagnosis, prognosis, resistance to anthracycline with taxane	Anti-VEGF therapies (e.g., bevacizumab)	[163]
	AURKA	Tumor tissue	Clinical, Phase I trial	Prognostic, resistance to tamoxifen, aromatase inhibitors, and endocrine resistance	AURKA inhibitors (e.g., alisertib) to overcome endocrine resistance	[164]
	RB1, ESR1, PTEN and KMT2C	Tumor biopsy and blood samples	Preclinical trial	Prognosis, resistance to CDK4/6 inhibitor, palbociclib with endocrine therapy	ESR1-targeted therapies or novel CDK inhibitors	[165]
ncRNA-based biomarkers	miR-195	Plasma, trastuzumab-resistant BT-474 cells	Preclinical	Diagnosis, trastuzumab resistance	miRNA-based therapeutics to restore trastuzumab sensitivity	[166,167]
	miR-1246	Plasma, trastuzumab-resistant BT-474 cells	Clinical trial	Diagnosis, trastuzumab resistance	miRNA-based diagnostics or therapeutics	[166]
	miR-155	Blood	Preclinical trial	Prognosis, resistance to trastuzumab	miR-155 inhibitors to enhance trastuzumab response	[168]
	miR-18a	Tumor tissue, MCF-7, MDA-MB-231	Preclinical	Prognosis, resistance to endocrine therapy	miR-18a inhibitors to restore endocrine sensitivity	[169]
	miR-20a-5p	Tumor tissue	Preclinical	Prognosis, diagnosis, resistance to chemotherapy	miR-20a-5p inhibitors to enhance chemotherapy response	[170]
	miR-139-5p	Solid tumor tissue, MCF-7	Preclinical	Prognosis, resistance to radiotherapy, PARP inhibitor, rucaparib, mitomycin C, cisplatin, doxorubicin	miR-139-5p mimics to sensitize to radiotherapy and chemotherapy	[171]
	miR-92a-3p	Serum, tumor tissue, MCF-7, MCF-7 derived tamoxifen-resistant cells	Preclinical	Prognosis, diagnosis, resistance to tamoxifen	miR-92a-3p inhibitors to overcome tamoxifen resistance	[172,173]
	miR-34a and miR-125b	Plasma	Clinical trial, phase III	Prognosis, diagnosis, resistance to neoadjuvant chemotherapy (anthracycline and taxane)	miRNA-based diagnostics for chemotherapy response	[174,175]
	miR-126 and miR-10a	Tumor tissue	Case control, observational	Prognosis, resistance to adjuvant tamoxifen	miRNA-targeted therapies to enhance tamoxifen efficacy	[176,177]
	LINK-A	Solid and metastatic tumor	Preclinical	Prognosis, diagnosis, resistance to Akt inhibitors and immunotherapy	LINK-A inhibitors to enhance Akt inhibitor response	[178,179]
	GAS5	Tumor tissue, SK-BR-3-, SK-BR-3-derived trastuzumab-resistant cells	Preclinical	Prognostic, targeting trastuzumab resistance	GAS5 mimics to restore trastuzumab sensitivity	[180]
	H19	Tumor tissue, SK-BR-3, HCC1954, MDA-MB-231	Preclinical	Prognosis, mediate trastuzumab resistance	H19 inhibitors to overcome trastuzumab resistance	[181]

ACLY: ATP citrate lyase; OS: overall survival; CDK4/6: cyclin-dependent kinase 4/6; AR-V7: androgen receptor splice variant 7; FASN: fatty-acid synthase; ncRNA: non-coding RNA; miRNA: microRNA.

## THE COMPLEX INTERPLAY OF TUMOR IMMUNE MICROENVIRONMENT AND TREATMENT RESISTANCE IN BREAST CANCER

The tumor immune microenvironment (TIME) is composed of diverse immune cells contributing to acquired therapeutic resistance in breast cancer. The acquired resistance is facilitated by the co-existence of cellular crosstalk between the tumor core and stromal components within the TME. An array of immune components contributes to multiple therapy resistance through various redundant mechanisms triggered by cancer cells, as depicted in Table 2.

**Table 2:** The molecular mechanism of immune cell-mediated drug resistance in breast cancer

**Therapeutic approach**	**Drug**	**Signaling involved**	**Source (cells/tissue)**	**Response**	**Ref.**
**TAMs**					
Endocrine therapy	Tamoxifen	PI3K/Akt/mTOR	MCF-7	Secrete CCL2	[182]
		HIF-1α/STAT3	MCF-7, T47D	M2 phenotype polarization	[183]
		NF-κB/STAT3/ERK	MCF-7	Secrete IL-6 and TNFα	[184]
	Aromatase inhibitors	Jagged1/Notch	THP-1, MCF-7	M2 phenotype polarization	[185]
		High expression of CXCR4	MCF-7, RAW264.7	Enhance the motility	[186]
Chemotherapy	Doxorubicin	NF-κB Ap-1(c-jun)	RAW264.7 MCF-7	Secrete IL-6	[187]
		PTEN/Akt	THP-1, MCF-7	M2 phenotype polarization	[188]
		IL-10R/STAT3/Bcl-2	MDA-MB-453, BT-20	Secrete IL-10	[189]
	Paclitaxel	IL-10/STAT3/Bcl-2	T47D, BT549	Secrete IL-10	[190]
		Insulin/IGF1R	4T1	Secrete IGF	[191]
	Carboplatin	GJIC	MDA-MB-231, T47D	Direct cell-cell interactions	[192]
Targeted therapy	Lapatinib	Src/STAT3/ERK1/2-mediated EGFR signaling	SK-BR-3	Secrete IL-8	[193]
	Trastuzumab	Upregulated expression of B7-H4	Bone marrow-derived macrophages, Peripheral blood mononuclear cells	Phenotype indicating immunosuppression	[194]
	PI3K inhibitor-GDC-0941	NF-κB	4T1	Secrete chemokines and cytokines	[195,196]
Immunotherapy	Anti-PD-1 antibody	High TYRO3 expression	4T1	Lower the M1/M2 TAM ratio	[197]
	CTLA-4 inhibitor	MSP-RON	F4/80+ peritoneal macrophages	PD-L1 expression is high	[198]
**NK cells**					
Targeted therapy	Trastuzumab	JAK2/STAT1	SK-BR-3, peripheral blood mononuclear cells	Elevate the expression of PD-1 on NK cells and PD-L1 on tumor cells	[199]
Chemotherapy	Taxanes-anthracyclines	Unclear	Patient-derived tumor tissue	Decreased NK cell infiltration into tumor tissue	[200]
**T cells**					
Chemotherapy	Paclitaxel	JAK-STAT3/MAPKs/Akt	MDA-MB-468, MDA-MB-231	Produce IL-22	[201]
Targeted therapy	Gefitinib	EGFR/IL-17E	MDA-MB-468	Produce IL-17E	[202,203]

TAMs: Tumor-associated macrophages; CCL2: CC-chemokine ligand 2; IGF: insulin-like growth factor; GJIC: gap junctional intercellular communication; NK: natural killer.

### Macrophage-mediated therapy resistance

One of the innate immune cells, macrophages, exerts dynamic phenotypic and functional changes in elevating treatment resistance, particularly due to the mutational landscape of cancer cells greatly influencing the response to therapy. For example, drug-resistant breast cancer cells secrete TNF-α, which triggers mTORC1-FOXK1 activation in TAMs and facilitates the M2 polarization of TAMs, enhancing the secretion of more CCL2 to create a positive feedback system. Furthermore, the PI3K/Akt/mTOR signaling pathway is activated by TAM-derived CCL2 in breast cancer cells, leading to increased TAM recruitment and enhanced breast cancer cell resistance^[182]^. Similarly, TAM-secreted TNF-α and IL-6 stimulate the NF-κB/STAT3/ERK signaling cascade in breast cancer cells. This leads to ERα hyperphosphorylation and the overexpression of IL-6, Cyclin D1, c-Myc, and CCL2 (MCP-1), resulting in enhanced tamoxifen resistance^[184]^. Moreover, TNF-α from inflammatory macrophages reduces the expression of ERα and activates the Akt pathway, leading to the phosphorylation of FOXO3a in breast cancer cells^[204]^. M2-like TAMs trigger the EGFR/PI3K/Akt signaling axis, leading to increased expression of sodium/glucose cotransporter 1 (SGLT1), which in turn induces resistance to tamoxifen in ER+ breast cancer cells^[183]^. Tamoxifen resistance is linked to elevated EGFR expression with an increased TAM infiltration in breast cancer tissues. This suggests that the crosstalk between TAMs and breast cancer cells exhibiting higher EGFR expression may play a role in the development of tamoxifen resistance^[205]^. The overexpression of IGF-1 and -2 in TAMs promotes the insulin/IGF1R signaling in TNBC cells. Research indicates that paclitaxel resistance may be caused by IGF expression or activation of the insulin/IGF-1 receptor^[191]^. Prior studies on LncRNAs revealed that LINC00337 enhances the oncogenic properties of breast cancer cells, thereby increasing resistance to paclitaxel via M2-like macrophages^[206]^. Olaparib, a PARP inhibitor, targets TNBC and regulates TAM function and metabolism. TAM recruitment increases, but CSF1R and PD-L1 are overexpressed. TAMs treated with olaparib reduce T cell proliferation and antitumor activity^[207]^. A study found that TAMs release IL-8, which activates EGFR signaling mediated by Src/STAT3/ERK1/2 in breast cancer cells and contributes to lapatinib resistance in HER2-positive breast cancer cells^[193]^. For recurring or metastatic breast cancer, bevacizumab is always administered alongside chemotherapy. TAMs polarize into the M2b type via the Fc-γ receptor and the TLR4 signaling pathways. By producing TNFα and triggering the immunosuppressive factor IDO1, M2b-type TAMs increase resistance to the treatment of bevacizumab^[208]^. Other reports suggested that the hypoxia-induced breast cancer cells release oncostatin M and eotaxin, which facilitate macrophage recruitment and polarization, promote angiogenesis in breast tumors, and lead to bevacizumab resistance^[209]^. Sorafenib therapy leads to enhanced accumulation and infiltration of M2-type TAMs in 4T1 tumors, resulting in breast tumor progression, while targeting TAMs reduces tumor proliferation^[210]^. In addition, TAMs also contribute to immunotherapy resistance in breast cancer^[211]^. For instance, breast tumors with elevated TYRO3 expression decrease tumor ferroptosis and sustain an immunosuppressive environment by lowering the M1/M2 TAMs ratio, leading to anti-PD-1 treatment resistance^[197]^. In addition, a recent study related to the BET inhibitor found that TAMs triggered by TNBC increase resistance to BET inhibitors via IL-6 or IL-10/STAT3/IKBKE/NF-κB signaling^[212]^. Research indicates that the interactions between TAMs and T cells, as well as the expression of PD-1/PD-L1, are associated with resistance to immunotherapy^[213,214]^.

### Natural killer cell-mediated immune resistance

It has been demonstrated that the innate immune system, which includes neutrophils, macrophages, antigen-presenting cells (APCs), and natural killer (NK) cells, has antitumor effects independent of adaptive immunity^[215]^. A favorable prognosis for TNBC is associated with an increased infiltration of NK cells that exhibit cytotoxic properties. NK cells are only seen in about 5% of breast cancer tumor-infiltrating lymphocytes (TILs), with the TNBC subtype showing higher levels of infiltration^[216]^. Since NK cells do not possess T cell receptors (TCRs), they are unable to identify tumor cells via the MHC-I molecule. Alternatively, they can target cancerous cells that are MHC-deficient^[217]^. This primary cytotoxic strategy supports adaptive immunity and provides additional treatment options for ICB therapy-resistant cancer patients due to adaptive mutations^[218]^. NK cells can also modulate the secretion of cytokines and chemokines alongside cytotoxicity. The release of IFN-γ boosts dendritic cell maturation, helper T cell activity, and MHC-I expression on tumor cells, making them more susceptible to T cells. NK cells can inhibit T cell activation by enhancing PD-L1 and LAG-3 expression and promoting angiogenesis, thereby facilitating immunological escape. Tumors with significant NK infiltration typically have limited T cell infiltration^[219]^.

### T cell-mediated immune tolerance

Within the TIME, the most prevalent cytotoxic lymphocytes are T cells, and tumor cells primarily activate them to increase immunogenicity. APCs present tumor-specific antigens (TSA), activating and recruiting T lymphocytes that destroy cancer cells^[215]^. T lymphocytes are prevented from over-activating and targeting healthy cells by PD1/PD-L1 inhibitory co-stimulatory signals under normal physiological parameters^[220]^. In addition, depletion of T cells can result from tumor cells overexpressing PD1/PD-L1, allowing them to evade immunological response. Likewise, T cell exhaustion in breast cancer, especially in TNBC and HER2-positive types, is identified by a gradual decline in T cell functionality as a result of prolonged antigen exposure. This condition is characterized by an increase in the expression of inhibitory receptors such as PD-1, CTLA-4, and TIM-3. T cells that have become exhausted show a diminished capacity to proliferate and generate effector cytokines, ultimately leading to weakened antitumor responses^[221]^. Breast cancer immunotherapy relies heavily on PD1/PD-L1 pathway blockers, such as PD-1 and PD-L1 monoclonal antibodies. Aberrant PD-L1 expression can result from abnormalities in genome transcription and translation, affecting immune function through aberrant signaling pathways. Excessive pro-inflammatory cytokines such as IFN-γ and TNF-α in the TME can facilitate immune escape by inducing PD-L1 expression in tumor cells via various signaling pathways^[220]^. Similarly, Myeloid-derived suppressor cells (MDSCs) gather in the TME and implement immunosuppressive actions by generating arginase, ROS, and various cytokines that inhibit T cell activation and function. In TNBC, MDSCs have been associated with resistance to chemotherapy, as they can create an environment conducive to tumor growth and reduce the cytotoxic impact of treatment^[222]^. Checkpoint adaptation is another crucial mechanism that allows breast cancer to evade the immune system. In HER2-positive breast cancer, tumors can adaptively increase PD-L1 expression on their surface, which can help the tumor evade T cell-mediated cytotoxicity, ultimately contributing to chemoresistance^[223]^. Additionally, METTL3 enhances PD-L1 expression in breast cancer cells through a post-transcriptional mechanism that is dependent on m6A-IGF2BP3. This process promotes mRNA stability and impacts the effectiveness of tumor immunotherapy^[224]^. Recent studies indicate that combining immune checkpoint inhibitors (ICIs) with traditional therapies may improve therapeutic outcomes in both TNBC and HER2-positive subtypes. This combination approach aims to rejuvenate exhausted T cells and counteract the immunosuppressive effects of MDSCs, thereby addressing the challenges of chemoresistance and enhancing patient outcomes^[225]^.

## CANCER IMMUNOTHERAPEUTICS TO OVERCOME BREAST TUMOR RESISTANCE

Breast tumors exhibit a complex array of pathways that trigger their resistance to immunotherapeutic approaches, and perplexing treatment outcomes. Tumor cell heterogeneity, which leads to diverse antigen expression and immune evasion, is a major contributing factor to this resistance. FOXP3+ Tregs inhibit antitumor responses, while ablation of FOXP3+ Tregs dramatically reduces tumor growth^[226,227]^. Additionally, certain malignancies undergo genetic alterations that affect the APCs, thereby preventing them from immune surveillance^[228]^. Another crucial component of tumor resistance is the overexpression of checkpoint molecules, including PD-L1, which can suppress T cell responses even when activated immune cells are present^[229]^.

The involvement of glycosylation in immune cell interactions is one of the many biological elements that greatly influence the intricacy of breast tumor resistance. Specifically, abnormal glycosylation of T lymphocytes directly impacts their ability to detect and kill tumor cells since these alterations can alter cell communication and immune activation dynamics^[230]^. Recent studies have emphasized the potential of combination therapies that pair ICIs with engineered immune cells to strengthen the immune response against breast cancer cells. For instance, the combination of endogenous PD-1 checkpoint inhibitors with chimeric antigen receptor T cells (CAR-T cells) holds the potential to enhance therapeutic efficacy by targeting tumor-associated antigens unique to breast cancer^[231-233]^. Cheng *et al.* have reported that T cell depletion is typically triggered by the PD-1/PD-L1 interaction, cytotoxicity suppression, and downregulation of the antitumor immune response^[232]^. Thus, combining CAR-T treatment with PD-1/PD-L1 inhibitors can improve T cell-mediated antitumor outcomes more than targeting LAG-3 or TIM-3^[232]^. In the past few years, the prognostic significance of TILs in metastatic breast malignancies has been investigated. In patients with advanced HER2-positive breast cancer treated with trastuzumab, docetaxel, and pertuzumab, elevated levels of TILs are notably linked to enhanced overall survival (OS)^[234]^. Furthermore, the use of dendritic cell vaccines promotes a strong T cell response, leading to long-term immune suppression against tumors. As research advances, the use of these approaches not only offers a tool to combat tumor resistance but also expands the range of treatments available to patients with breast cancer, paving the way for better prognostic results^[235]^.

## CLINICAL IMPLICATIONS OF MOLECULAR FINDINGS TOWARD ONGOING TRANSLATIONAL THERAPEUTIC ADVANCES

Recent clinical studies have offered valuable insights into overcoming treatment resistance by focusing on molecular and epigenetic modifications, paving the way for combination therapies and biomarker-guided approaches^[105]^. Preclinical studies have provided critical mechanistic insights into breast cancer resistance, identifying genetic and epigenetic drivers such as 3D epigenome remodeling and clonal heterogeneity^[236]^. Tools such as CRISPR/Cas9 and single-cell epigenomics have identified precise targets (e.g., ESR1, PTEN, PD-L1), revealing mechanisms such as PTEN loss, CDK4/6 upregulation, and PD-L1 regulation^[237,238]^.

In trastuzumab-resistant HER2-positive breast cancer, the EMILIA Trial (NCT00829166) demonstrated that trastuzumab emtansine increases progression-free survival (PFS)^[239]^. The E2112 Trial (NCT02115282) evaluated the combination of entinostat and exemestane in ER+ metastatic breast cancer, showing a slight improvement in PFS but limited OS benefits^[240]^. The Phase II Trial (NCT01349959) of azacitidine combined with entinostat in TNBC showed limited clinical efficacy, despite considering epigenetic changes (e.g., reduced methylation), indicating that better results could be achieved with higher dosages or combinatorial immunotherapy^[241]^. The combination of neratinib and capecitabine in the TBCRC 022 Trial (NCT01494662) showed intracranial action in brain metastases of HER2-positive breast cancer, suggesting the possibility of bilateral HER2 pathway inhibition in resistant scenarios^[242]^. The DEBBRAH trial (NCT04420598) showed trastuzumab deruxtecan’s effectiveness for brain metastases in HER2-low advanced breast cancer, with a 41.7% response rate and acceptable toxicity, requiring further research^[243]^. A Phase II trial (NCT01022138) demonstrated that anti-CD3 × anti-HER2 bispecific antibody armed activated T cells (HER2 BATs) exposure after chemotherapy in case of metastatic breast cancer improves the quality of life and OS^[244]^. The MONARCH 2 Trial (NCT02107703) showed that ctDNA-based biomarkers can identify patients with PIK3CA and ESR1 mutations who may not respond well to certain treatments, confirming the value of liquid biopsy for monitoring resistance^[245]^. A Phase I Trial (NCT02167854) investigating alpelisib in combination with HER3 inhibitors for metastatic breast cancer demonstrated disease control but was limited by gastrointestinal toxicity^[246]^. Phase II Trial (NCT02910050) combined bicalutamide with aromatase inhibitors in resistant ER+/AR+ breast cancer, showing no synergy or objective responses^[247]^. Ongoing trials are incorporating epigenetic biomarkers to bridge preclinical and clinical insights^[248]^. Although clinical trials validate therapeutic efficacy, they often lack epigenetic biomarkers, limiting mechanistic insights. Combining epigenetic therapies with immunotherapy or targeted therapies holds synergistic potential, as suggested by preclinical studies^[248]^. However, limitations in preclinical models, such as inadequate TME modeling and *in vitro* artifacts, hinder direct clinical translation of these findings. These findings underscore the need to integrate multi-omics profiling (genomics, epigenomics, and transcriptomics) to inform clinical trial design^[249]^.

The advancement of single-cell sequencing, spatial transcriptomics, and AI technologies has improved the understanding of breast cancer research, revealing different molecular types and ways to overcome treatment resistance^[250]^. Spatial transcriptomics can help pinpoint changes in the immune system, surrounding cells, and blood vessels, aiding personalized treatment strategies. Single-cell epigenetic sequencing examines inheritable information related to cell structures and gene expression^[251]^. Single-cell sequencing is transforming breast cancer resistance profiling by revealing tumor heterogeneity with unparalleled precision, overcoming limitations of bulk sequencing^[252]^. It facilitates the detection of resistant subclones before clinical resistance manifestation, enabling early intervention^[253]^. In HER2-positive breast cancer, this technology has investigated combinatorial immunotherapy strategies for CDK4/6 inhibitor resistance by elucidating rapidly evolving resistance mechanisms^[254]^. Additionally, it provides comprehensive transcriptomic insights into lymph node metastasis and neoadjuvant chemotherapy-resistant TNBC^[255,256]^. By enabling dynamic, patient-specific resistance profiling, it paves the way for tailored therapeutic strategies in precision oncology^[250]^. Thus, innovative technologies are recognized as essential instruments for interpreting TME heterogeneity and customizing treatment approaches, including single-cell sequencing, spatial transcriptomics, and nanomedicine. TME dynamics are influenced by predictive spatial modeling to predict treatment outcomes. GeoMx DSP measures stromal and immunological markers in TME and finds spatial correlations between immune markers with resistance and survival mechanisms (such as VISTA). AI enhances diagnosis and prognosis by integrating various data types, such as genomics, medical images, and electronic records, to create precise models of tumor resistance. It can also develop predictions about how tumors respond to different drugs, optimize combinations, and analyze features related to drug resistance. AI is utilized in evaluating drug sensitivity through various models. PDOs are used for high-throughput drug sensitivity screening and to replicate patient-specific genetic and phenotypic tumors. An AI-powered spatial analyzer of TILs has recently shown that the spatial distribution of TILs helps explain key immune phenotypes, such as immune-excluded and inflamed types. These phenotypes are strongly linked to tumor mutational burden, responsiveness to ICIs, and patient survival. This highlights the importance of AI analytics in improving the therapeutic prediction^[257,258]^.

AI improves cancer diagnosis and prognosis by integrating diverse data sources such as genomics and medical images to predict tumor drug responses and optimize treatments, but faces challenges in data bias, interpretability, and standardized data management^[257,258]^. While advancements have been made, ongoing improvements in technology are necessary for better integration of AI and multi-omics in clinical oncology, ultimately enabling more effective personalized treatments.

## CONCLUSION AND FUTURE PERSPECTIVES

**Figure 4:**
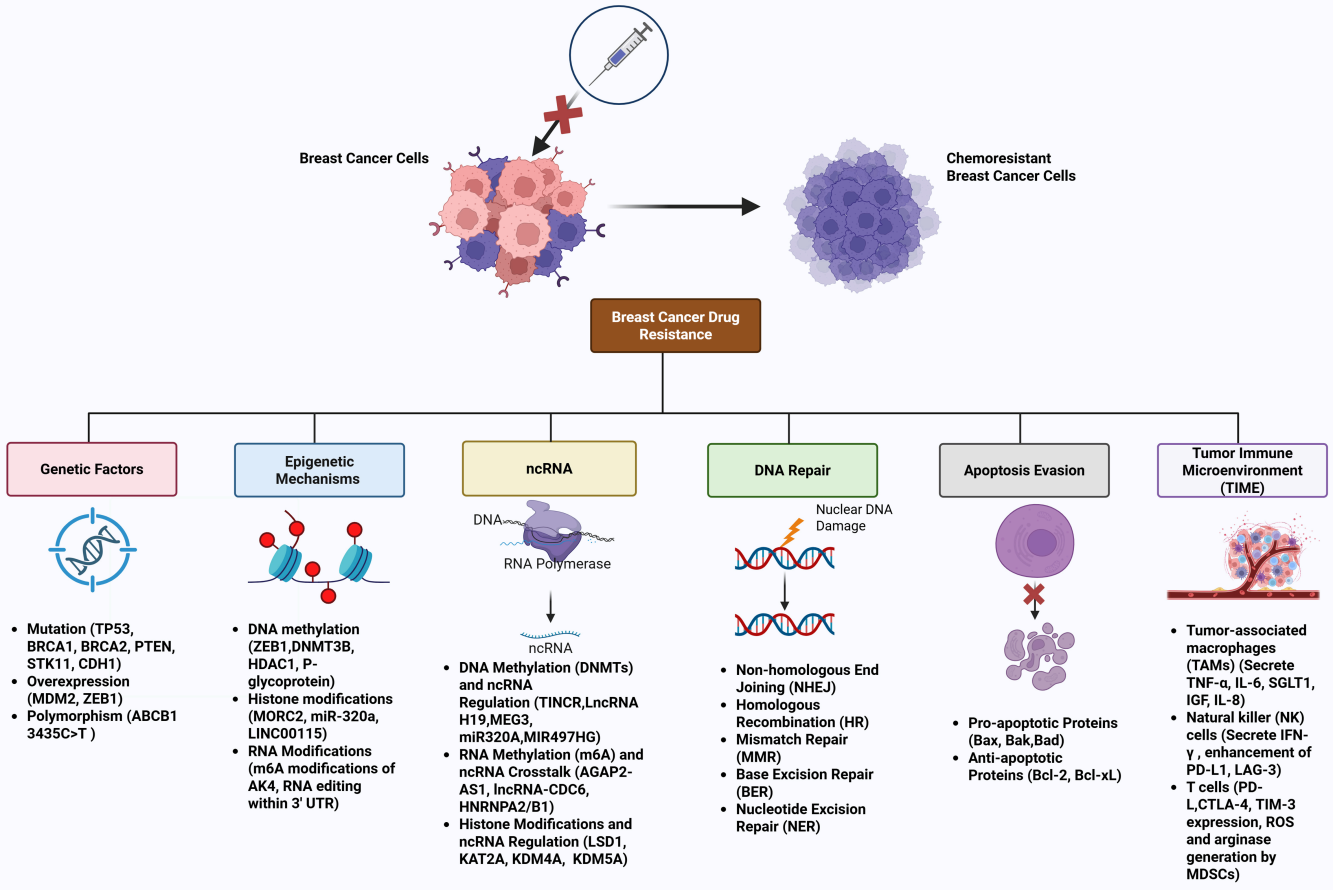
Major drivers of breast cancer therapeutic resistance. This schematic diagram illustrates the multifaceted mechanisms underlying therapeutic resistance in breast cancer, encompassing genetic factors, epigenetic mechanisms, ncRNAs, apoptosis evasion, DNA repair, and the TIME. Genetic Factors, such as oncogenic mutations (e.g., PTEN, TP53) and amplifications (e.g., HER2, MDM2), activate proliferative pathways like PI3K/AKT/mTOR, conferring resistance to chemotherapies and targeted therapies. Epigenetic mechanisms, involving aberrant DNA methylation by DNMTs and histone modifications (e.g., EZH2-mediated H3K27me3, LSD1-driven demethylation), silence tumor suppressors (e.g., PTEN, GREB1), promoting therapeutic resistance. lncRNAs (e.g., HOTAIR, MALAT1, CDC6) and miRNAs (e.g., miR-320a, miR-21) modulate oncogenic signaling via m6A modifications, enhancing EMT and chemoresistance. Furthermore, upregulation of anti-apoptotic proteins (e.g., Bcl-2) and downregulation of pro-apoptotic factors (e.g., Bax) via epigenetic regulation impede chemotherapy-induced cell death. In addition, activation of robust repair pathways (e.g., HR, NHEJ, BER, NER, MMR) in resistant cells counteracts chemotherapy-induced DNA damage (e.g., DSBs, crosslinks). Moreover, immunosuppressive elements, including T cell exhaustion (PD-1/CTLA-4 upregulation), MDSC infiltration, and checkpoint adaptation (PD-L1 overexpression), create a resistant TME, particularly in TNBC and HER2-positive subtypes. The interplay of these drivers underscores the complexity of therapeutic resistance, highlighting potential targets for precision oncology. Created in BioRender. Malhotra, D. (2025) s1b4tq4. ncRNAs: Non-coding RNAs; TIME: tumor immune microenvironment; MDM2: mouse double minute 2; GREB1: growth-regulating estrogen receptor binding 1; HR: homologous recombination; NHEJ: non-homologous end joining; BER: base excision repair; NER: nucleotide excision repair; MMR: mismatch repair; DSBs: double-strand breaks; TME: tumor microenvironment; TNBC: triple-negative breast cancer.

Breast cancer treatment resistance poses a substantial obstacle caused by genetic mutations, epigenetic changes in gene expression, and immune-regulatory adaptations. The interplay of these elements leads to tumor heterogeneity, evasion of immune responses, and treatment failures, especially in aggressive forms such as triple-negative and HER2-positive breast cancers. The most predominant drivers of breast cancer therapeutic resistance are summarized in Figure 4, which include a complex interplay of genetic factors (e.g., PTEN, TP53 mutations, HER2 amplification), epigenetic mechanisms (e.g., DNMT-mediated methylation, EZH2-driven H3K27me3), ncRNAs (e.g., HOTAIR, miR-21 with m6A modifications), apoptosis evasion (e.g., Bcl-2 upregulation), robust DNA repair (e.g., HR, NHEJ), and an immunosuppressive TIME (e.g., T cell exhaustion, MDSC infiltration, PD-L1 overexpression), particularly in TNBC and HER2-positive subtypes, highlighting multifaceted targets for precision oncology.

Although important advancements have been achieved in understanding crucial molecular pathways, there are still notable gaps in knowledge and ongoing controversies. One significant gap involves the insufficient comprehension of how genetic evolution and epigenetic plasticity interact over time under therapy-induced selection pressure. The debate over whether resistance mainly exists prior to treatment or develops *de novo* complicates efforts to administer targeted therapies. Moreover, the interaction between RNA methylation (m6A) and ncRNAs in influencing drug resistance is a promising new area of research, but its clinical implications and potential for therapeutic application need further validation. A promising future direction is the use of combined approaches that analyze different types of genetic data to identify specific resistance mechanisms in patients. This may help develop customized treatments for various types of breast cancer. For instance, Butti *et al.* have successfully developed patient-derived orthotopic xenografts (PDOXs) from hormone-resistant breast cancer patients to study various genes involved in drug resistance^[259]^. In a similar line, epigenetic treatments including agents targeting DNA methylation (e.g., decitabine), histone modifications (e.g., HDAC inhibitors), and RNA methylation (m6A modulators) are emerging as beneficial complements to chemotherapy and immunotherapy^[61,77]^. The synergy of these agents with ICIs, such as anti-PD-1/PD-L1 therapies, may enhance immune responses against tumors, particularly in cases involving epigenetic silencing of immunity-related genes^[260]^. Furthermore, in the field of ncRNA therapeutics, abnormally expressed ncRNAs often affect changes in gene expression and facilitate immune evasion in resistant breast cancer forms^[261]^. Developing advanced delivery systems, such as nanoparticle-based carriers, to convey lncRNA/miRNA mimics or inhibitors directly into tumor cells may assist in altering resistance mechanisms and restoring treatment sensitivity. It will be essential to tackle issues concerning the stability, specificity, and off-target effects of ncRNA therapies for clinical use, with current investigations into lipid-based and exosome-mediated delivery approaches offering more promising prospects^[262]^.

Focusing on immune evasion strategies is another critical area of interest. Resistant breast cancers often exhibit T cell exhaustion and are infiltrated by MDSCs, which impede antitumor immune responses^[263]^. Next-generation ICIs targeting novel markers (e.g., LAG-3, TIGIT) and therapies aimed at depleting or reprogramming MDSCs may rejuvenate immune responses^[264]^. Tailored immunotherapy regimens, informed by immune profiling of the TME using methods such as single-cell sequencing and spatial transcriptomics, could improve response rates by aligning treatment to specific immune contexts^[265]^. These cutting-edge technologies facilitate thorough mapping of immune cell types and their interactions within tumors, paving the way for the development of targeted immunotherapeutic approaches. Moreover, advancements in single-cell sequencing, spatial transcriptomics, and AI-enhanced predictive models will accelerate the journey toward precision medicine. Single-cell sequencing reveals intratumor heterogeneity and identifies resistant subclones, while spatial transcriptomics provides insights into the location of resistant cells within the tumor milieu^[257,265]^. AI models trained on multi-omics datasets are capable of predicting resistance trends and recommending patient-specific treatment strategies, thus supporting clinical decision making^[257]^. Moreover, tumor-treating fields, combined with chemotherapy, disrupt mitotic proteins by employing alternating electric fields in aggressive brain tumors, with conducting probe atomic force microscopy (C-AFM)-derived chromosomal electron flux data revealing mechanisms to enhance therapeutic outcomes^[266]^. Chromosome-based bioelectronics holds promise for innovative medical device applications in precision oncology. These technologies will also facilitate the discovery of novel biomarkers, promoting the early detection of resistance and timely modifications to treatment plans. In this context, adaptive clinical trials that evaluate multi-target therapies, updated clinical guidelines that incorporate insights into resistance mechanisms, and policies that ensure equitable access to advanced diagnostic technologies such as next-generation sequencing will be vital. By fostering collaborative interdisciplinary efforts and employing bioinformatics to synthesize complex data, this review envisions a future where innovative therapies, enhanced diagnostic tools, and personalized approaches effectively tackle the persistent challenge of treatment resistance in breast cancer, ultimately improving patient outcomes worldwide.

## DECLARATIONS

### Copyright

© The Author(s) 2025.

## References

[1] Giaquinto AN, Sung H, Newman LA (2024). Breast cancer statistics 2024. CA Cancer J Clin.

[2] Zhao H (2021). The prognosis of invasive ductal carcinoma, lobular carcinoma and mixed ductal and lobular carcinoma according to molecular subtypes of the breast. Breast Cancer.

[3] Łukasiewicz S, Czeczelewski M, Forma A, Baj J, Sitarz R, Stanisławek A (2021). Breast cancer-epidemiology, risk factors, classification, prognostic markers, and current treatment strategies-an updated review. Cancers.

[4] Arps DP, Healy P, Zhao L, Kleer CG, Pang JC (2013). Invasive ductal carcinoma with lobular features: a comparison study to invasive ductal and invasive lobular carcinomas of the breast. Breast Cancer Res Treat.

[5] Waks AG, Winer EP (2019). Breast cancer treatment: a review. JAMA.

[6] Mandapati A, Lukong KE (2023). Triple negative breast cancer: approved treatment options and their mechanisms of action. J Cancer Res Clin Oncol.

[7] Nikolaou M, Pavlopoulou A, Georgakilas AG, Kyrodimos E (2018). The challenge of drug resistance in cancer treatment: a current overview. Clin Exp Metastasis.

[8] Bhushan A, Gonsalves A, Menon JU (2021). Current state of breast cancer diagnosis, treatment, and theranostics. Pharmaceutics.

[9] Hayes DF, Isaacs C, Stearns V (2001). Prognostic factors in breast cancer: current and new predictors of metastasis. J Mammary Gland Biol Neoplasia.

[10] Weigel MT, Dowsett M (2010). Current and emerging biomarkers in breast cancer: prognosis and prediction. Endocr Relat Cancer.

[11] Zhou Y, Tao L, Qiu J (2024). Tumor biomarkers for diagnosis, prognosis and targeted therapy. Signal Transduct Target Ther.

[12] Nalejska E, Mączyńska E, Lewandowska MA (2014). Prognostic and predictive biomarkers: tools in personalized oncology. Mol Diagn Ther.

[13] Wu HJ, Chu PY (2021). Recent discoveries of macromolecule- and cell-based biomarkers and therapeutic implications in breast cancer. Int J Mol Sci.

[14] Dagogo-Jack I, Shaw AT (2018). Tumour heterogeneity and resistance to cancer therapies. Nat Rev Clin Oncol.

[15] Sun XX, Yu Q (2015). Intra-tumor heterogeneity of cancer cells and its implications for cancer treatment. Acta Pharmacol Sin.

[16] (2023). Visser KE, Joyce JA. The evolving tumor microenvironment: from cancer initiation to metastatic outgrowth. Cancer Cell.

[17] Lavie D, Ben-Shmuel A, Erez N, Scherz-Shouval R (2022). Cancer-associated fibroblasts in the single-cell era. Nat Cancer.

[18] Butti R, Kundu GC (2023). The molecular dialogue between the tumor cells and fibroblasts. Oncotarget.

[19] Yuan J, Liu M, Yang L (2015). Acquisition of epithelial-mesenchymal transition phenotype in the tamoxifen-resistant breast cancer cell: a new role for G protein-coupled estrogen receptor in mediating tamoxifen resistance through cancer-associated fibroblast-derived fibronectin and β1-integrin signaling pathway in tumor cells. Breast Cancer Res.

[20] Mao Y, Zhang Y, Qu Q (2015). Cancer-associated fibroblasts induce trastuzumab resistance in HER2 positive breast cancer cells. Mol Biosyst.

[21] Gao Y, Li X, Zeng C (2020). CD63^+^ cancer-associated fibroblasts confer tamoxifen resistance to breast cancer cells through exosomal miR-22. Adv Sci.

[22] Su S, Chen J, Yao H (2018). CD10^+^GPR77^+^ cancer-associated fibroblasts promote cancer formation and chemoresistance by sustaining cancer stemness. Cell.

[23] Fan G, Yu B, Tang L (2024). TSPAN8^+^ myofibroblastic cancer-associated fibroblasts promote chemoresistance in patients with breast cancer. Sci Transl Med.

[24] Du R, Zhang X, Lu X (2023). PDPN positive CAFs contribute to HER2 positive breast cancer resistance to trastuzumab by inhibiting antibody-dependent NK cell-mediated cytotoxicity. Drug Resist Updat.

[25] Radharani NNV, Yadav AS, Nimma R (2022). Tumor-associated macrophage derived IL-6 enriches cancer stem cell population and promotes breast tumor progression via Stat-3 pathway. Cancer Cell Int.

[26] Huang R, Kang T, Chen S (2024). The role of tumor-associated macrophages in tumor immune evasion. J Cancer Res Clin Oncol.

[27] Phi LTH, Sari IN, Yang YG (2018). Cancer stem cells (CSCs) in drug resistance and their therapeutic implications in cancer treatment. Stem Cells Int.

[28] Alison MR, Lim SM, Nicholson LJ (2011). Cancer stem cells: problems for therapy?. J Pathol.

[29] Dean M, Fojo T, Bates S (2005). Tumour stem cells and drug resistance. Nat Rev Cancer.

[30] Zheng Q, Zhang M, Zhou F, Zhang L, Meng X (2020). The breast cancer stem cells traits and drug resistance. Front Pharmacol.

[31] Bourguignon LY, Peyrollier K, Xia W, Gilad E (2008). Hyaluronan-CD44 interaction activates stem cell marker Nanog, Stat-3-mediated MDR1 gene expression, and ankyrin-regulated multidrug efflux in breast and ovarian tumor cells. J Biol Chem.

[32] Sansone P, Ceccarelli C, Berishaj M (2016). Self-renewal of CD133^hi^ cells by IL6/Notch3 signalling regulates endocrine resistance in metastatic breast cancer. Nat Commun.

[33] Wang T, Gantier MP, Xiang D (2015). EpCAM aptamer-mediated survivin silencing sensitized cancer stem cells to doxorubicin in a breast cancer model. Theranostics.

[34] Croker AK, Allan AL (2012). Inhibition of aldehyde dehydrogenase (ALDH) activity reduces chemotherapy and radiation resistance of stem-like ALDHhiCD44^+^ human breast cancer cells. Breast Cancer Res Treat.

[35] Pal M, Das D, Pandey M (2024). Understanding genetic variations associated with familial breast cancer. World J Surg Oncol.

[36] Kennedy RD, Quinn JE, Mullan PB, Johnston PG, Harkin DP (2004). The role of BRCA1 in the cellular response to chemotherapy. J Natl Cancer Inst.

[37] Steelman LS, Navolanic PM, Sokolosky ML (2008). Suppression of PTEN function increases breast cancer chemotherapeutic drug resistance while conferring sensitivity to mTOR inhibitors. Oncogene.

[38] Wu H, Kang H, Liu Y (2012). Roles of ABCB1 gene polymorphisms and haplotype in susceptibility to breast carcinoma risk and clinical outcomes. J Cancer Res Clin Oncol.

[39] Tulsyan S, Mittal RD, Mittal B (2016). The effect of ABCB1 polymorphisms on the outcome of breast cancer treatment. Pharmgenomics Pers Med.

[40] Geisler S, Lonning PE, Aas T, Johnsen H, Fluge O, Haugen DF, Lillehaug JR, Akslen LA, Borresen-Dale AL (2001). Influence of TP53 gene alterations and c-erbB-2 expression on the response to treatment with doxorubicin in locally advanced breast cancer. Cancer Res.

[41] Geisler S, Borresen-Dale AL, Johnsen H, Aas T, Geisler J, Akslen LA, Anker G, Lonning PE (2003). TP53 gene mutations predict the response to neoadjuvant treatment with 5-fluorouracil and mitomycin in locally advanced breast cancer. Clin Cancer Res.

[42] Chrisanthar R, Knappskog S, Løkkevik E (2008). CHEK2 mutations affecting kinase activity together with mutations in TP53 indicate a functional pathway associated with resistance to epirubicin in primary breast cancer. PLoS One.

[43] Hou H, Sun D, Zhang X (2019). The role of MDM2 amplification and overexpression in therapeutic resistance of malignant tumors. Cancer Cell Int.

[44] Zhang X, Zhang Z, Zhang Q (2018). ZEB1 confers chemotherapeutic resistance to breast cancer by activating ATM. Cell Death Dis.

[45] Prabhu KS, Sadida HQ, Kuttikrishnan S, Junejo K, Bhat AA, Uddin S (2024). Beyond genetics: exploring the role of epigenetic alterations in breast cancer. Pathol Res Pract.

[46] Roulois D, Loo Yau H, Singhania R (2015). DNA-demethylating agents target colorectal cancer cells by inducing viral mimicry by endogenous transcripts. Cell.

[47] Deblois G, Tonekaboni SAM, Grillo G (2020). Epigenetic switch-induced viral mimicry evasion in chemotherapy-resistant breast cancer. Cancer Discov.

[48] Wajapeyee N, Gupta R (2021). Epigenetic alterations and mechanisms that drive resistance to targeted cancer therapies. Cancer Res.

[49] Yadav M, Vaishkiar I, Sharma A (2024). Oestrogen receptor positive breast cancer and its embedded mechanism: breast cancer resistance to conventional drugs and related therapies, a review. Open Biol.

[50] Sukocheva OA, Lukina E, Friedemann M, Menschikowski M, Hagelgans A, Aliev G (2022). The crucial role of epigenetic regulation in breast cancer anti-estrogen resistance: current findings and future perspectives. Semin Cancer Biol.

[51] Romero-Garcia S, Prado-Garcia H, Carlos-Reyes A (2020). Role of DNA methylation in the resistance to therapy in solid tumors. Front Oncol.

[52] Zhang J, Zhou C, Jiang H (2017). ZEB1 induces ER-α promoter hypermethylation and confers antiestrogen resistance in breast cancer. Cell Death Dis.

[53] Chekhun VF, Kulik GI, Yurchenko OV (2006). Role of DNA hypomethylation in the development of the resistance to doxorubicin in human MCF-7 breast adenocarcinoma cells. Cancer Lett.

[54] Ansar M, Thu LTA, Hung CS (2022). Promoter hypomethylation and overexpression of TSTD1 mediate poor treatment response in breast cancer. Front Oncol.

[55] Liu HY, Liu YY, Yang F (2020). Acetylation of MORC2 by NAT10 regulates cell-cycle checkpoint control and resistance to DNA-damaging chemotherapy and radiotherapy in breast cancer. Nucleic Acids Res.

[56] He DX, Gu XT, Jiang L, Jin J, Ma X (2014). A methylation-based regulatory network for microRNA 320a in chemoresistant breast cancer. Mol Pharmacol.

[57] Luo F, Zhang M, Sun B (2024). LINC00115 promotes chemoresistant breast cancer stem-like cell stemness and metastasis through SETDB1/PLK3/HIF1α signaling. Mol Cancer.

[58] Kumari K, Groza P, Aguilo F (2021). Regulatory roles of RNA modifications in breast cancer. NAR Cancer.

[59] Song H, Liu D, Dong S (2020). Epitranscriptomics and epiproteomics in cancer drug resistance: therapeutic implications. Signal Transduct Target Ther.

[60] Liu X, Gonzalez G, Dai X (2020). Adenylate kinase 4 modulates the resistance of breast cancer cells to tamoxifen through an m^6^A-based epitranscriptomic mechanism. Mol Ther.

[61] Wang J, Xu J, Zheng J (2023). A1BG-AS1 promotes adriamycin resistance of breast cancer by recruiting IGF2BP2 to upregulate ABCB1 in an m6A-dependent manner. Sci Rep.

[62] Nakano M, Fukami T, Gotoh S, Nakajima M (2017). A-to-I RNA editing up-regulates human dihydrofolate reductase in breast cancer. J Biol Chem.

[63] Millán-Zambrano G, Burton A, Bannister AJ, Schneider R (2022). Histone post-translational modifications - cause and consequence of genome function. Nat Rev Genet.

[64] Mattei AL, Bailly N, Meissner A (2022). DNA methylation: a historical perspective. Trends Genet.

[65] Zhang G, Cheng C, Wang X, Wang S (2024). N6-Methyladenosine methylation modification in breast cancer: current insights. J Transl Med.

[66] Wang Q, Liu J, You Z (2021). LncRNA TINCR favors tumorigenesis via STAT3-TINCR-EGFR-feedback loop by recruiting DNMT1 and acting as a competing endogenous RNA in human breast cancer. Cell Death Dis.

[67] Wang Q, Li G, Ma X (2023). LncRNA TINCR impairs the efficacy of immunotherapy against breast cancer by recruiting DNMT1 and downregulating MiR-199a-5p via the STAT1-TINCR-USP20-PD-L1 axis. Cell Death Dis.

[68] Wang J, Xie S, Yang J (2019). The long noncoding RNA H19 promotes tamoxifen resistance in breast cancer via autophagy. J Hematol Oncol.

[69] He DX, Ma X (2016). Transient receptor potential channel C5 in cancer chemoresistance. Acta Pharmacol Sin.

[70] Pan T, Ding H, Jin L (2022). DNMT1-mediated demethylation of lncRNA MEG3 promoter suppressed breast cancer progression by repressing Notch1 signaling pathway. Cell Cycle.

[71] Huang ZF, Tang YL, Shen ZL, Yang KY, Gao K (2022). UXT, a novel DNMT3b-binding protein, promotes breast cancer progression via negatively modulating lncRNA MEG3/p53 axis. Mol Ther Oncolytics.

[72] Si X, Zang R, Zhang E (2016). LncRNA H19 confers chemoresistance in ERα-positive breast cancer through epigenetic silencing of the pro-apoptotic gene BIK. Oncotarget.

[73] Tian Y, Chen ZH, Wu P (2023). MIR497HG-derived miR-195 and miR-497 mediate tamoxifen resistance via PI3K/AKT signaling in breast cancer. Adv Sci.

[74] Cai Y, Zheng H, Xu D (2024). M6A RNA methylation-mediated dysregulation of AGAP2-AS1 promotes trastuzumab resistance of breast cancer. Pharmacology.

[75] Zhang H, Wang J, Liu C, Yan K, Wang X, Sheng X (2025). Interactions between long non-coding RNAs and m6 A modification in cancer. Discov Oncol.

[76] Liu X, Xie X, Sui C (2024). Unraveling the cross-talk between N6-methyladenosine modification and non-coding RNAs in breast cancer: mechanisms and clinical implications. Int J Cancer.

[77] Wang D, Han Y, Peng L (2023). Crosstalk between N6-methyladenosine (m6A) modification and noncoding RNA in tumor microenvironment. Int J Biol Sci.

[78] Chen T, Zheng L, Luo P (2024). Crosstalk between m6A modification and autophagy in cancer. Cell Biosci.

[79] Klinge CM, Piell KM, Tooley CS, Rouchka EC (2019). HNRNPA2/B1 is upregulated in endocrine-resistant LCC9 breast cancer cells and alters the miRNA transcriptome when overexpressed in MCF-7 cells. Sci Rep.

[80] Wu Y, Zhang Z, Cenciarini ME (2018). Tamoxifen resistance in breast cancer is regulated by the EZH2-ERα-GREB1 transcriptional axis. Cancer Res.

[81] Kim MR, Wu MJ, Zhang Y, Yang JY, Chang CJ (2020). TET2 directs mammary luminal cell differentiation and endocrine response. Nat Commun.

[82] Oh JH, Lee JY, Kim KH (2020). Elevated GCN5 expression confers tamoxifen resistance by upregulating AIB1 expression in ER-positive breast cancer. Cancer Lett.

[83] Boulding T, McCuaig RD, Tan A (2018). LSD1 activation promotes inducible EMT programs and modulates the tumour microenvironment in breast cancer. Sci Rep.

[84] Choi HJ, Joo HS, Won HY (2018). Role of RBP2-induced ER and IGF1R-ErbB signaling in tamoxifen resistance in breast cancer. J Natl Cancer Inst.

[85] Zhou Q, Song C, Liu X, Qin H, Miao L, Zhang X (2019). Peptidylarginine deiminase 4 overexpression resensitizes MCF-7/ADR breast cancer cells to adriamycin via GSK3β/p53 activation. Cancer Manag Res.

[86] Kaboli PJ, Rahmat A, Ismail P, Ling KH (2015). MicroRNA-based therapy and breast cancer: a comprehensive review of novel therapeutic strategies from diagnosis to treatment. Pharmacol Res.

[87] Kaboli PJ, Imani S, Jomhori M, Ling KH (2021). Chemoresistance in breast cancer: PI3K/Akt pathway inhibitors vs the current chemotherapy. Am J Cancer Res.

[88] Li Y, Zhai Z, Li H, Wang X, Huang Y, Su X (2019). Guajadial reverses multidrug resistance by inhibiting ABC transporter expression and suppressing the PI3K/Akt pathway in drug-resistant breast cancer cells. Chem Biol Interact.

[89] Qian J, Xia M, Liu W (2019). Glabridin resensitizes p-glycoprotein-overexpressing multidrug-resistant cancer cells to conventional chemotherapeutic agents. Eur J Pharmacol.

[90] McGlynn LM, Kirkegaard T, Edwards J (2009). Ras/Raf-1/MAPK pathway mediates response to tamoxifen but not chemotherapy in breast cancer patients. Clin Cancer Res.

[91] Saatci O, Huynh-Dam KT, Sahin O (2021). Endocrine resistance in breast cancer: from molecular mechanisms to therapeutic strategies. J Mol Med.

[92] Bai WD, Ye XM, Zhang MY (2014). MiR-200c suppresses TGF-β signaling and counteracts trastuzumab resistance and metastasis by targeting ZNF217 and ZEB1 in breast cancer. Int J Cancer.

[93] Palomeras S, Diaz-Lagares Á, Viñas G (2019). Epigenetic silencing of TGFBI confers resistance to trastuzumab in human breast cancer. Breast Cancer Res.

[94] Bartucci M, Dattilo R, Moriconi C (2015). TAZ is required for metastatic activity and chemoresistance of breast cancer stem cells. Oncogene.

[95] Donninger H, Vos MD, Clark GJ (2007). The RASSF1A tumor suppressor. J Cell Sci.

[96] Zeng R, Dong J (2021). The Hippo signaling pathway in drug resistance in cancer. Cancers.

[97] Liu L, Tommasi S, Lee DH, Dammann R, Pfeifer GP (2003). Control of microtubule stability by the RASSF1A tumor suppressor. Oncogene.

[98] Er I, Boz Er AB (2024). Hedgehog pathway is a regulator of stemness in HER2-positive trastuzumab-resistant breast cancer. Int J Mol Sci.

[99] Wils LJ, Bijlsma MF (2018). Epigenetic regulation of the Hedgehog and Wnt pathways in cancer. Crit Rev Oncol Hematol.

[100] Gyamfi J, Lee YH, Eom M, Choi J (2020). Author Correction: Interleukin-6/STAT3 signalling regulates adipocyte induced epithelial-mesenchymal transition in breast cancer cells. Sci Rep.

[101] Xing J, Li J, Fu L, Gai J, Guan J, Li Q (2019). SIRT4 enhances the sensitivity of ER-positive breast cancer to tamoxifen by inhibiting the IL-6/STAT3 signal pathway. Cancer Med.

[102] Kettner NM, Vijayaraghavan S, Durak MG (2019). Combined inhibition of STAT3 and DNA repair in palbociclib-resistant ER-positive breast cancer. Clin Cancer Res.

[103] Xiang S, Dauchy RT, Hoffman AE (2019). Epigenetic inhibition of the tumor suppressor ARHI by light at night-induced circadian melatonin disruption mediates STAT3-driven paclitaxel resistance in breast cancer. J Pineal Res.

[104] Ye L, Lin C, Wang X (2019). Epigenetic silencing of SALL2 confers tamoxifen resistance in breast cancer. EMBO Mol Med.

[105] Garcia-Martinez L, Zhang Y, Nakata Y, Chan HL, Morey L (2021). Epigenetic mechanisms in breast cancer therapy and resistance. Nat Commun.

[106] O’Brien CS, Howell SJ, Farnie G, Clarke RB (2009). Resistance to endocrine therapy: are breast cancer stem cells the culprits?. J Mammary Gland Biol Neoplasia.

[107] Butti R, Das S, Gunasekaran VP, Yadav AS, Kumar D, Kundu GC (2018). Receptor tyrosine kinases (RTKs) in breast cancer: signaling, therapeutic implications and challenges. Mol Cancer.

[108] Nami B, Ghanaeian A, Black C, Wang Z (2021). Epigenetic silencing of HER2 expression during epithelial-mesenchymal transition leads to trastuzumab resistance in breast cancer. Life.

[109] Jackson SP, Bartek J (2009). The DNA-damage response in human biology and disease. Nature.

[110] Jurkovicova D, Neophytou CM, Gašparović AČ, Gonçalves AC (2022). DNA damage response in cancer therapy and resistance: challenges and opportunities. Int J Mol Sci.

[111] Wang S, Zou Z, Luo X, Mi Y, Chang H, Xing D (2018). LRH1 enhances cell resistance to chemotherapy by transcriptionally activating MDC1 expression and attenuating DNA damage in human breast cancer. Oncogene.

[112] Tu X, Kahila MM, Zhou Q (2018). ATR inhibition is a promising radiosensitizing strategy for triple-negative breast cancer. Mol Cancer Ther.

[113] Luo M, Kelley MR (2004). Inhibition of the human apurinic/apyrimidinic endonuclease (APE1) repair activity and sensitization of breast cancer cells to DNA alkylating agents with lucanthone. Anticancer Res.

[114] Neophytou CM, Trougakos IP, Erin N, Papageorgis P (2021). Apoptosis deregulation and the development of cancer multi-drug resistance. Cancers.

[115] Abbas R, Larisch S (2020). Targeting XIAP for promoting cancer cell death-the story of ARTS and SMAC. Cells.

[116] Ozaki T, Nakagawara A (2011). Role of p53 in cell death and human cancers. Cancers.

[117] Du K, Montminy M (1998). CREB is a regulatory target for the protein kinase Akt/PKB. J Biol Chem.

[118] Liu R, Chen Y, Liu G (2020). PI3K/AKT pathway as a key link modulates the multidrug resistance of cancers. Cell Death Dis.

[119] Safa AR (2022). Drug and apoptosis resistance in cancer stem cells: a puzzle with many pieces. Cancer Drug Resist.

[120] Breunig C, Pahl J, Küblbeck M (2017). MicroRNA-519a-3p mediates apoptosis resistance in breast cancer cells and their escape from recognition by natural killer cells. Cell Death Dis.

[121] Sun WL, Lan D, Gan TQ, Cai ZW (2015). Autophagy facilitates multidrug resistance development through inhibition of apoptosis in breast cancer cells. Neoplasma.

[122] Townsend PA, Kozhevnikova MV, Cexus ONF, Zamyatnin AA Jr, Soond SM (2021). BH3-mimetics: recent developments in cancer therapy. J Exp Clin Cancer Res.

[123] Bose P, Gandhi V, Konopleva M (2017). Pathways and mechanisms of venetoclax resistance. Leuk Lymphoma.

[124] Neizer-Ashun F, Bhattacharya R (2021). Reality CHEK: understanding the biology and clinical potential of CHK1. Cancer Lett.

[125] Fuino L, Bali P, Wittmann S, Donapaty S, Guo F, Yamaguchi H, Wang HG, Atadja P, Bhalla K (2003). Histone deacetylase inhibitor LAQ824 down-regulates Her-2 and sensitizes human breast cancer cells to trastuzumab, taxotere, gemcitabine, and epothilone B. Mol Cancer Ther.

[126] Li ZH, Weng X, Xiong QY (2017). miR-34a expression in human breast cancer is associated with drug resistance. Oncotarget.

[127] Yang R, Stöcker S, Schott S (2017). The association between breast cancer and S100P methylation in peripheral blood by multicenter case-control studies. Carcinogenesis.

[128] Hannafon BN, Trigoso YD, Calloway CL (2016). Plasma exosome microRNAs are indicative of breast cancer. Breast Cancer Res.

[129] Iliopoulos D, Jaeger SA, Hirsch HA, Bulyk ML, Struhl K (2010). STAT3 activation of miR-21 and miR-181b-1 via PTEN and CYLD are part of the epigenetic switch linking inflammation to cancer. Mol Cell.

[130] Iorio MV, Ferracin M, Liu CG (2005). MicroRNA gene expression deregulation in human breast cancer. Cancer Res.

[131] Han Y, Wang J, Sun T (2023). Predictive biomarkers of response and survival following immunotherapy with a PD-L1 inhibitor benmelstobart (TQB2450) and antiangiogenic therapy with a VEGFR inhibitor anlotinib for pretreated advanced triple negative breast cancer. Signal Transduct Target Ther.

[132] Lei JH, Zhang L, Wang Z (2022). FGFR2-BRD4 axis regulates transcriptional networks of histone 3 modification and synergy between its inhibitors and PD-1/PD-L1 in a TNBC mouse model. Front Immunol.

[133] Voulgarelis D, Forment JV, Herencia Ropero A (2024). Understanding tumour growth variability in breast cancer xenograft models identifies PARP inhibition resistance biomarkers. NPJ Precis Oncol.

[134] Sha LY, Zhang Y, Wang W, Sui X, Liu SK, Wang T, Zhang H (2016). MiR-18a upregulation decreases Dicer expression and confers paclitaxel resistance in triple negative breast cancer. Eur Rev Med Pharmacol Sci.

[135] Nair MG, Prabhu JS, Korlimarla A (2020). miR-18a activates Wnt pathway in ER-positive breast cancer and is associated with poor prognosis. Cancer Med.

[136] Ling Y, Liang G, Lin Q (2022). circCDYL2 promotes trastuzumab resistance via sustaining HER2 downstream signaling in breast cancer. Mol Cancer.

[137] Siouda M, Dujardin AD, Barbollat-Boutrand L (2020). CDYL2 epigenetically regulates MIR124 to control NF-κB/STAT3-dependent breast cancer cell plasticity. iScience.

[138] Chen Y, Li K, Gong D (2020). ACLY: a biomarker of recurrence in breast cancer. Pathol Res Pract.

[139] Chen D, Chen X, Yang M (2025). H3K36me2 methyltransferase NSD2/WHSC1 promotes triple-negative breast cancer metastasis via activation of ULK1-dependent autophagy. Autophagy.

[140] Gökmen-Polar Y, Neelamraju Y, Goswami CP (2015). Expression levels of SF3B3 correlate with prognosis and endocrine resistance in estrogen receptor-positive breast cancer. Mod Pathol.

[141] Guertin DA, Wellen KE (2023). Acetyl-CoA metabolism in cancer. Nat Rev Cancer.

[143] Kim GJ, Kim DH, Min KW, Kim YH, Oh YH (2018). Loss of p27(kip1) expression is associated with poor prognosis in patients with taxane-treated breast cancer. Pathol Res Pract.

[144] Wang J, Xu B, Yuan P (2012). TOP2A amplification in breast cancer is a predictive marker of anthracycline-based neoadjuvant chemotherapy efficacy. Breast Cancer Res Treat.

[146] Ginzac A, Molnar I, Durando X (2024). Neoadjuvant anthracycline-based (5-FEC) or anthracycline-free (docetaxel/carboplatin) chemotherapy plus trastuzumab and pertuzmab in HER2 + BC patients according to their TOP2A: a multicentre, open-label, non-randomized phase II trial. Breast Cancer Res Treat.

[147] Slamon DJ, Leyland-Jones B, Shak S (2001). Use of chemotherapy plus a monoclonal antibody against HER2 for metastatic breast cancer that overexpresses HER2. N Engl J Med.

[149] Cai R, Chen Q, Zhao D (2024). A high immune-related index with the suppression of cGAS-STING pathway is a key determinant to herceptin resistance in HER2+ breast cancer. Int J Biol Sci.

[150] Li H, Zhang M, Wei Y (2020). SH3BGRL confers innate drug resistance in breast cancer by stabilizing HER2 activation on cell membrane. J Exp Clin Cancer Res.

[151] Chen YC, Li HY, Liang JL (2017). CTMP, a predictive biomarker for trastuzumab resistance in HER2-enriched breast cancer patient. Oncotarget.

[152] Klintman M, Dowsett M (2015). Early surrogate markers of treatment activity: where are we now?. J Natl Cancer Inst Monogr.

[153] Elzawahry HM, Saber MM, Mokhtar NM, Zeeneldin AA, Ismail YM, Alieldin NH (2013). Role of Ki67 in predicting resistance to adjuvant tamoxifen in postmenopausal breast cancer patients. J Egypt Natl Canc Inst.

[154] Srivastava TP, Ajmeriya S, Goel I (2024). Prognostic role of androgen receptor splice variant 7 (AR-V7) in the pathogenesis of breast cancer. BMC Cancer.

[155] Warmoes M, Jaspers JE, Xu G (2013). Proteomics of genetically engineered mouse mammary tumors identifies fatty acid metabolism members as potential predictive markers for cisplatin resistance. Mol Cell Proteomics.

[156] Al-Bahlani S, Al-Lawati H, Al-Adawi M, Al-Abri N, Al-Dhahli B, Al-Adawi K (2017). Fatty acid synthase regulates the chemosensitivity of breast cancer cells to cisplatin-induced apoptosis. Apoptosis.

[157] Sardesai SD, Thomas A, Gallagher C (2021). Inhibiting fatty acid synthase with omeprazole to improve efficacy of neoadjuvant chemotherapy in patients with operable TNBC. Clin Cancer Res.

[159] Li K, Shu D, Li H (2024). SMAD4 depletion contributes to endocrine resistance by integrating ER and ERBB signaling in HR + HER2- breast cancer. Cell Death Dis.

[160] Finn RS, Martin M, Rugo HS (2016). Palbociclib and letrozole in advanced breast cancer. N Engl J Med.

[161] Denkert C, Seither F, Schneeweiss A (2021). Clinical and molecular characteristics of HER2-low-positive breast cancer: pooled analysis of individual patient data from four prospective, neoadjuvant clinical trials. Lancet Oncol.

[162] Grote I, Bartels S, Kandt L (2021). TP53 mutations are associated with primary endocrine resistance in luminal early breast cancer. Cancer Med.

[163] Miles DW, Chan A, Dirix LY (2010). Phase III study of bevacizumab plus docetaxel compared with placebo plus docetaxel for the first-line treatment of human epidermal growth factor receptor 2-negative metastatic breast cancer. J Clin Oncol.

[164] Haddad TC, D’Assoro A, Suman V (2018). Phase I trial to evaluate the addition of alisertib to fulvestrant in women with endocrine-resistant, ER+ metastatic breast cancer. Breast Cancer Res Treat.

[165] Park YH, Im SA, Park K (2023). Longitudinal multi-omics study of palbociclib resistance in HR-positive/HER2-negative metastatic breast cancer. Genome Med.

[166] Rezaei Z, Dastjerdi K, Allahyari A (2023). Plasma microRNA-195, -34c, and -1246 as novel biomarkers for the diagnosis of trastuzumab-resistant HER2-positive breast cancer patients. Toxicol Appl Pharmacol.

[167] Wang ZH, Zheng ZQ, Jia SC (2022). Trastuzumab resistance in HER2-positive breast cancer: mechanisms, emerging biomarkers and targeting agents. Front Oncol.

[168] Zhang Z, Zhang L, Yu G (2020). Exosomal miR-1246 and miR-155 as predictive and prognostic biomarkers for trastuzumab-based therapy resistance in HER2-positive breast cancer. Cancer Chemother Pharmacol.

[169] Luengo-Gil G, García-Martínez E, Chaves-Benito A (2019). Clinical and biological impact of miR-18a expression in breast cancer after neoadjuvant chemotherapy. Cell Oncol.

[170] Huang W, Wu X, Xiang S (2022). Regulatory mechanism of miR-20a-5p expression in cancer. Cell Death Discov.

[171] Pajic M, Froio D, Daly S (2018). miR-139-5p modulates radiotherapy resistance in breast cancer by repressing multiple gene networks of DNA repair and ROS defense. Cancer Res.

[172] Zou X, Xia T, Li M (2021). MicroRNA profiling in serum: potential signatures for breast cancer diagnosis. Cancer Biomark.

[173] Cun J, Yang Q (2018). Bioinformatics-based interaction analysis of miR-92a-3p and key genes in tamoxifen-resistant breast cancer cells. Biomed Pharmacother.

[174] Xiong H, Song Z, Wang T (2025). Photoswitchable dynamics and RNAi synergist with tailored interface and controlled release reprogramming tumor immunosuppressive niche. Biomaterials.

[175] Kassem NM, Makar WS, Kassem HA (2019). Circulating miR-34a and miR-125b as promising non invasive biomarkers in egyptian locally advanced breast cancer patients. Asian Pac J Cancer Prev.

[176] Hoppe R, Achinger-Kawecka J, Winter S (2013). Increased expression of miR-126 and miR-10a predict prolonged relapse-free time of primary oestrogen receptor-positive breast cancer following tamoxifen treatment. Eur J Cancer.

[177] Kahraman M, Röske A, Laufer T (2018). MicroRNA in diagnosis and therapy monitoring of early-stage triple-negative breast cancer. Sci Rep.

[178] Liao B, Wang J, Xie Y, Luo H, Min J (2024). LINK-A: unveiling its functional role and clinical significance in human tumors. Front Cell Dev Biol.

[179] Lin A, Hu Q, Li C (2017). The LINK-A lncRNA interacts with PtdIns(3,4,5)P_3_ to hyperactivate AKT and confer resistance to AKT inhibitors. Nat Cell Biol.

[180] Li W, Zhai L, Wang H (2016). Downregulation of LncRNA GAS5 causes trastuzumab resistance in breast cancer. Oncotarget.

[181] Sun Z, Zhang C, Wang T, Shi P, Tian X, Guo Y (2019). Correlation between long non-coding RNAs (lncRNAs) H19 expression and trastuzumab resistance in breast cancer. J Cancer Res Ther.

[182] Li D, Ji H, Niu X (2020). Tumor-associated macrophages secrete CC-chemokine ligand 2 and induce tamoxifen resistance by activating PI3K/Akt/mTOR in breast cancer. Cancer Sci.

[183] Niu X, Ma J, Li J (2021). Sodium/glucose cotransporter 1-dependent metabolic alterations induce tamoxifen resistance in breast cancer by promoting macrophage M2 polarization. Cell Death Dis.

[184] Castellaro AM, Rodriguez-Baili MC, Di Tada CE, Gil GA (2019). Tumor-associated macrophages induce endocrine therapy resistance in ER+ breast cancer cells. Cancers.

[185] Liu H, Wang J, Zhang M (2017). Jagged1 promotes aromatase inhibitor resistance by modulating tumor-associated macrophage differentiation in breast cancer patients. Breast Cancer Res Treat.

[186] Gelsomino L, Giordano C, Camera G (2020). Leptin signaling contributes to aromatase inhibitor resistant breast cancer cell growth and activation of macrophages. Biomolecules.

[187] Dong X, Sun R, Wang J (2020). Glutathione S-transferases P1-mediated interleukin-6 in tumor-associated macrophages augments drug-resistance in MCF-7 breast cancer. Biochem Pharmacol.

[188] Chen WX, Wang DD, Zhu B (2021). Exosomal miR-222 from adriamycin-resistant MCF-7 breast cancer cells promote macrophages M2 polarization via PTEN/Akt to induce tumor progression. Aging.

[189] Emami F, Pathak S, Nguyen TT (2021). Photoimmunotherapy with cetuximab-conjugated gold nanorods reduces drug resistance in triple negative breast cancer spheroids with enhanced infiltration of tumor-associated macrophages. J Control Release.

[190] Yang C, He L, He P (2015). Increased drug resistance in breast cancer by tumor-associated macrophages through IL-10/STAT3/bcl-2 signaling pathway. Med Oncol.

[191] Ireland L, Santos A, Campbell F (2018). Blockade of insulin-like growth factors increases efficacy of paclitaxel in metastatic breast cancer. Oncogene.

[192] Walker ND, Elias M, Guiro K (2019). Exosomes from differentially activated macrophages influence dormancy or resurgence of breast cancer cells within bone marrow stroma. Cell Death Dis.

[193] Ahmed S, Mohamed HT, El-Husseiny N (2021). IL-8 secreted by tumor associated macrophages contribute to lapatinib resistance in HER2-positive locally advanced breast cancer via activation of Src/STAT3/ERK1/2-mediated EGFR signaling. Biochim Biophys Acta Mol Cell Res.

[194] Hu X, Liu Y, Zhang X (2020). The anti-B7-H4 checkpoint synergizes trastuzumab treatment to promote phagocytosis and eradicate breast cancer. Neoplasia.

[195] Usman MW, Gao J, Zheng T (2019). Author Correction: Macrophages confer resistance to PI3K inhibitor GDC-0941 in breast cancer through the activation of NF-κB signaling. Cell Death Dis.

[196] Usman MW, Gao J, Zheng T (2018). Macrophages confer resistance to PI3K inhibitor GDC-0941 in breast cancer through the activation of NF-κB signaling. Cell Death Dis.

[197] Jiang Z, Lim SO, Yan M (2021). TYRO3 induces anti-PD-1/PD-L1 therapy resistance by limiting innate immunity and tumoral ferroptosis. J Clin Invest.

[198] Ekiz HA, Lai SA, Gundlapalli H, Haroun F, Williams MA, Welm AL (2018). Inhibition of RON kinase potentiates anti-CTLA-4 immunotherapy to shrink breast tumors and prevent metastatic outgrowth. Oncoimmunology.

[199] Zheng G, Guo Z, Li W (2021). Interaction between HLA-G and NK cell receptor KIR2DL4 orchestrates HER2-positive breast cancer resistance to trastuzumab. Signal Transduct Target Ther.

[200] Garcia-Chagollan M, Carranza-Torres IE, Carranza-Rosales P (2018). Expression of NK cell surface receptors in breast cancer tissue as predictors of resistance to antineoplastic treatment. Technol Cancer Res Treat.

[201] Wang S, Yao Y, Yao M, Fu P, Wang W (2018). Interleukin-22 promotes triple negative breast cancer cells migration and paclitaxel resistance through JAK-STAT3/MAPKs/AKT signaling pathways. Biochem Biophys Res Commun.

[202] Merrouche Y, Fabre J, Cure H (2016). IL-17E synergizes with EGF and confers in vitro resistance to EGFR-targeted therapies in TNBC cells. Oncotarget.

[203] Maniati E, Soper R, Hagemann T (2010). Up for Mischief? IL-17/Th17 in the tumour microenvironment. Oncogene.

[204] Gunnarsdóttir FB, Hagerling C, Bergenfelz C (2020). Inflammatory macrophage derived TNFα downregulates estrogen receptor α via FOXO3a inactivation in human breast cancer cells. Exp Cell Res.

[205] Xuan QJ, Wang JX, Nanding A (2014). Tumor-associated macrophages are correlated with tamoxifen resistance in the postmenopausal breast cancer patients. Pathol Oncol Res.

[206] Xing Z, Zhang M, Liu J, Liu G, Feng K, Wang X (2021). LINC00337 induces tumor development and chemoresistance to paclitaxel of breast cancer by recruiting M2 tumor-associated macrophages. Mol Immunol.

[207] Mehta AK, Cheney EM, Hartl CA (2021). Targeting immunosuppressive macrophages overcomes PARP inhibitor resistance in BRCA1-associated triple-negative breast cancer. Nat Cancer.

[208] Liu Y, Ji X, Kang N (2020). Tumor necrosis factor α inhibition overcomes immunosuppressive M2b macrophage-induced bevacizumab resistance in triple-negative breast cancer. Cell Death Dis.

[209] Tripathi C, Tewari BN, Kanchan RK (2014). Macrophages are recruited to hypoxic tumor areas and acquire a pro-angiogenic M2-polarized phenotype via hypoxic cancer cell derived cytokines Oncostatin M and Eotaxin. Oncotarget.

[210] Zhang C, Gao L, Cai Y (2016). Inhibition of tumor growth and metastasis by photoimmunotherapy targeting tumor-associated macrophage in a sorafenib-resistant tumor model. Biomaterials.

[211] Nagano M, Saito K, Kozuka Y (2021). CD204-positive macrophages accumulate in breast cancer tumors with high levels of infiltrating lymphocytes and programmed death ligand-1 expression. Oncol Lett.

[212] Qiao J, Chen Y, Mi Y (2020). Macrophages confer resistance to BET inhibition in triple-negative breast cancer by upregulating IKBKE. Biochem Pharmacol.

[213] Qin J, Zhang X, Tan B (2020). Blocking P2X7-mediated macrophage polarization overcomes treatment resistance in lung cancer. Cancer Immunol Res.

[214] Tan B, Shi X, Zhang J (2018). Inhibition of Rspo-Lgr4 facilitates checkpoint blockade therapy by switching macrophage polarization. Cancer Res.

[215] Yang YL, Yang F, Huang ZQ (2023). T cells, NK cells, and tumor-associated macrophages in cancer immunotherapy and the current state of the art of drug delivery systems. Front Immunol.

[216] Li R, Cao L (2023). The role of tumor-infiltrating lymphocytes in triple-negative breast cancer and the research progress of adoptive cell therapy. Front Immunol.

[217] Wu X, Li T, Jiang R, Yang X, Guo H, Yang R (2023). Targeting MHC-I molecules for cancer: function, mechanism, and therapeutic prospects. Mol Cancer.

[218] Zheng Y, Li S, Tang H, Meng X, Zheng Q (2023). Molecular mechanisms of immunotherapy resistance in triple-negative breast cancer. Front Immunol.

[219] Jorgovanovic D, Song M, Wang L, Zhang Y (2020). Roles of IFN-γ in tumor progression and regression: a review. Biomark Res.

[220] Lin X, Kang K, Chen P (2024). Regulatory mechanisms of PD-1/PD-L1 in cancers. Mol Cancer.

[221] Nair R, Somasundaram V, Kuriakose A (2025). Deciphering T-cell exhaustion in the tumor microenvironment: paving the way for innovative solid tumor therapies. Front Immunol.

[222] He S, Zheng L, Qi C (2025). Myeloid-derived suppressor cells (MDSCs) in the tumor microenvironment and their targeting in cancer therapy. Mol Cancer.

[223] Padmanabhan R, Kheraldine HS, Meskin N, Vranic S, Al Moustafa AE (2020). Crosstalk between HER2 and PD-1/PD-L1 in breast cancer: from clinical applications to mathematical models. Cancers.

[224] Wan W, Ao X, Chen Q (2022). METTL3/IGF2BP3 axis inhibits tumor immune surveillance by upregulating N^6^-methyladenosine modification of PD-L1 mRNA in breast cancer. Mol Cancer.

[225] Ji Q, Ding J, Hao M, Luo N, Huang J, Zhang W (2021). Immune checkpoint inhibitors combined with chemotherapy compared with chemotherapy alone for triple-negative breast cancer: a systematic review and meta-analysis. Front Oncol.

[226] Li C, Jiang P, Wei S, Xu X, Wang J (2020). Regulatory T cells in tumor microenvironment: new mechanisms, potential therapeutic strategies and future prospects. Mol Cancer.

[227] Esquivel-Velázquez M, Ostoa-Saloma P, Palacios-Arreola MI, Nava-Castro KE, Castro JI, Morales-Montor J (2015). The role of cytokines in breast cancer development and progression. J Interferon Cytokine Res.

[228] Zhang S, Xiao X, Yi Y (2024). Tumor initiation and early tumorigenesis: molecular mechanisms and interventional targets. Signal Transduct Target Ther.

[229] Han Y, Liu D, Li L (2020). PD-1/PD-L1 pathway: current researches in cancer. Am J Cancer Res.

[230] (2020). De Bousser E, Meuris L, Callewaert N, Festjens N. Human T cell glycosylation and implications on immune therapy for cancer. Hum Vaccin Immunother.

[231] Chen N, Morello A, Tano Z, Adusumilli PS (2017). CAR T-cell intrinsic PD-1 checkpoint blockade: a two-in-one approach for solid tumor immunotherapy. Oncoimmunology.

[232] Cheng K, Feng X, Chai Z (2023). 4-1BB-based CAR T cells effectively reverse exhaustion and enhance the anti-tumor immune response through autocrine PD-L1 scFv antibody. Int J Mol Sci.

[233] Hu W, Zi Z, Jin Y (2019). CRISPR/Cas9-mediated PD-1 disruption enhances human mesothelin-targeted CAR T cell effector functions. Cancer Immunol Immunother.

[234] Loi S, Sirtaine N, Piette F (2013). Prognostic and predictive value of tumor-infiltrating lymphocytes in a phase III randomized adjuvant breast cancer trial in node-positive breast cancer comparing the addition of docetaxel to doxorubicin with doxorubicin-based chemotherapy: BIG 02-98. J Clin Oncol.

[235] Gautam N, Ramamoorthi G, Champion N, Han HS, Czerniecki BJ (2024). Reviewing the significance of dendritic cell vaccines in interrupting breast cancer development. Mol Aspects Med.

[236] Casado-Pelaez M, Bueno-Costa A, Esteller M (2022). Single cell cancer epigenetics. Trends Cancer.

[237] Pont M, Marqués M, Sorolla MA (2023). Applications of CRISPR technology to breast cancer and triple negative breast cancer research. Cancers.

[238] Karn V, Sandhya S, Hsu W (2022). CRISPR/Cas9 system in breast cancer therapy: advancement, limitations and future scope. Cancer Cell Int.

[240] Connolly RM, Zhao F, Miller KD (2021). E2112: randomized Phase III Trial of endocrine therapy plus entinostat or placebo in hormone receptor-positive advanced breast cancer. A Trial of the ECOG-ACRIN Cancer Research Group. J Clin Oncol.

[241] Li H, Chiappinelli KB, Guzzetta AA (2014). Immune regulation by low doses of the DNA methyltransferase inhibitor 5-azacitidine in common human epithelial cancers. Oncotarget.

[242] Freedman RA, Heiling HM, Li T (2024). Neratinib and ado-trastuzumab emtansine for pretreated and untreated human epidermal growth factor receptor 2 (HER2)-positive breast cancer brain metastases: Translational Breast Cancer Research Consortium trial 022. Ann Oncol.

[243] Vaz Batista M, Pérez-García JM, Cortez P (2024). Trastuzumab deruxtecan in patients with previously treated HER2-low advanced breast cancer and active brain metastases: the DEBBRAH trial. ESMO Open.

[244] Lum LG, Al-Kadhimi Z, Deol A (2021). Phase II clinical trial using anti-CD3 × anti-HER2 bispecific antibody armed activated T cells (HER2 BATs) consolidation therapy for HER2 negative (0-2+) metastatic breast cancer. J Immunother Cancer.

[245] Tolaney SM, Toi M, Neven P (2022). Correction: Clinical significance of PIK3CA and ESR1 mutations in circulating tumor DNA: analysis from the MONARCH 2 study of abemaciclib plus fulvestrant. Clin Cancer Res.

[246] Jhaveri K, Drago JZ, Shah PD (2021). A phase I study of alpelisib in combination with trastuzumab and LJM716 in patients with PIK3CA-mutated HER2-positive metastatic breast cancer. Clin Cancer Res.

[247] Lu Q, Xia W, Lee K (2020). Bicalutamide plus aromatase inhibitor in patients with estrogen receptor-positive/androgen receptor-positive advanced breast cancer. Oncologist.

[248] Brown LJ, Achinger-Kawecka J, Portman N, Clark S, Stirzaker C, Lim E (2022). Epigenetic therapies and biomarkers in breast cancer. Cancers.

[249] Fountzilas E, Tsimberidou AM, Vo HH, Kurzrock R (2022). Clinical trial design in the era of precision medicine. Genome Med.

[250] Ren L, Li J, Wang C (2021). Single cell RNA sequencing for breast cancer: present and future. Cell Death Discov.

[251] Li M, Yan T, Wang M, Cai Y, Wei Y (2022). Advances in single-cell sequencing technology and its applications in triple-negative breast cancer. Breast Cancer.

[252] Chung W, Eum HH, Lee HO (2017). Single-cell RNA-seq enables comprehensive tumour and immune cell profiling in primary breast cancer. Nat Commun.

[253] Kim C, Gao R, Sei E (2018). Chemoresistance evolution in triple-negative breast cancer delineated by single-cell sequencing. Cell.

[254] Wang Q, Guldner IH, Golomb SM (2019). Single-cell profiling guided combinatorial immunotherapy for fast-evolving CDK4/6 inhibitor-resistant HER2-positive breast cancer. Nat Commun.

[255] Xu K, Wang R, Xie H (2021). Single-cell RNA sequencing reveals cell heterogeneity and transcriptome profile of breast cancer lymph node metastasis. Oncogenesis.

[256] Vishnubalaji R, Alajez NM (2021). Transcriptional landscape associated with TNBC resistance to neoadjuvant chemotherapy revealed by single-cell RNA-seq. Mol Ther Oncolytics.

[257] Mao Y, Shangguan D, Huang Q (2025). Emerging artificial intelligence-driven precision therapies in tumor drug resistance: recent advances, opportunities, and challenges. Mol Cancer.

[258] Yu J, Kong X, Feng Y (2025). Tumor microenvironment-driven resistance to immunotherapy in non-small cell lung cancer: strategies for Cold-to-Hot tumor transformation. Cancer Drug Resist.

[259] Butti R, Kapse P, Bhadauriya G (2023). Development and characterization of a patient-derived orthotopic xenograft of therapy-resistant breast cancer. Oncol Rep.

[260] Heater NK, Warrior S, Lu J (2024). Current and future immunotherapy for breast cancer. J Hematol Oncol.

[261] Saadi W, Fatmi A, Pallardó FV, García-Giménez JL, Mena-Molla S (2022). Long non-coding RNAs as epigenetic regulators of immune checkpoints in cancer immunity. Cancers.

[262] Grillone K, Caridà G, Luciano F (2024). A systematic review of non-coding RNA therapeutics in early clinical trials: a new perspective against cancer. J Transl Med.

[263] Tom VV, Jose AM, Mallick S (2025). Current advancement of immune function paradox of tumour-infiltrating cells and their immunotherapeutic targets: a mini-review. Naunyn Schmiedebergs Arch Pharmacol.

[264] Liu D, Liu L, Zhao X (2025). A comprehensive review on targeting diverse immune cells for anticancer therapy: beyond immune checkpoint inhibitors. Crit Rev Oncol Hematol.

[265] Xiong X, Wang X, Liu CC, Shao ZM, Yu KD (2024). Deciphering breast cancer dynamics: insights from single-cell and spatial profiling in the multi-omics era. Biomark Res.

[266] Agrawal T, Paul D, Mishra A, Arunkumar G, Rakshit T (2025). Epigenetic modifier drug valproic acid enhances cancer metaphase chromosome elasticity and electron transport: an atomic force microscopy approach. JACS Au.

